# Characterization of Nontypable *Haemophilus influenzae* Isolates Recovered from Adult Patients with Underlying Chronic Lung Disease Reveals Genotypic and Phenotypic Traits Associated with Persistent Infection

**DOI:** 10.1371/journal.pone.0097020

**Published:** 2014-05-13

**Authors:** Junkal Garmendia, Cristina Viadas, Laura Calatayud, Joshua Chang Mell, Pau Martí-Lliteras, Begoña Euba, Enrique Llobet, Carmen Gil, José Antonio Bengoechea, Rosemary J. Redfield, Josefina Liñares

**Affiliations:** 1 Instituto de Agrobiotecnología, CSIC-Universidad Pública Navarra-Gobierno Navarra, Mutilva, Spain; 2 Centro de Investigación Biomédica en Red de Enfermedades Respiratorias (CIBERES), Madrid, Spain; 3 Laboratory Microbial Pathogenesis, Fundación Investigación Sanitaria Illes Balears, Bunyola, Spain; 4 Microbiology Department, University Hospital Bellvitge, IDIBELL, University of Barcelona, Barcelona, Spain; 5 Department of Zoology, University British Columbia, Vancouver, British Columbia, Canada; 6 Department of Microbiology and Immunology, Drexel University College of Medicine, Philadelphia, Pennsylvania, United States of America; 7 Consejo Superior de Investigaciones Científicas (CSIC), Madrid, Spain; INIAV, I.P. National Institute of Agriculture and Veterinary Research, Portugal

## Abstract

Nontypable *Haemophilus influenzae* (NTHi) has emerged as an important opportunistic pathogen causing infection in adults suffering obstructive lung diseases. Existing evidence associates chronic infection by NTHi to the progression of the chronic respiratory disease, but specific features of NTHi associated with persistence have not been comprehensively addressed. To provide clues about adaptive strategies adopted by NTHi during persistent infection, we compared sequential persistent isolates with newly acquired isolates in sputa from six patients with chronic obstructive lung disease. Pulse field gel electrophoresis (PFGE) identified three patients with consecutive persistent strains and three with new strains. Phenotypic characterisation included infection of respiratory epithelial cells, bacterial self-aggregation, biofilm formation and resistance to antimicrobial peptides (AMP). Persistent isolates differed from new strains in showing low epithelial adhesion and inability to form biofilms when grown under continuous-flow culture conditions in microfermenters. Self-aggregation clustered the strains by patient, not by persistence. Increasing resistance to AMPs was observed for each series of persistent isolates; this was not associated with lipooligosaccharide decoration with phosphorylcholine or with lipid A acylation. Variation was further analyzed for the series of three persistent isolates recovered from patient 1. These isolates displayed comparable growth rate, natural transformation frequency and murine pulmonary infection. Genome sequencing of these three isolates revealed sequential acquisition of single-nucleotide variants in the AMP permease *sapC*, the heme acquisition systems *hgpB*, *hgpC*, *hup* and *hxuC*, the 3-deoxy-D-manno-octulosonic acid kinase *kdkA*, the long-chain fatty acid transporter *ompP1*, and the phosphoribosylamine glycine ligase *purD*. Collectively, we frame a range of pathogenic traits and a repertoire of genetic variants in the context of persistent infection by NTHi.

## Introduction

Chronic respiratory diseases including chronic obstructive pulmonary disease (COPD), bronchiectasis or cystic fibrosis (CF) often progress with lower airways colonization by pathogenic microorganisms and, in many cases, chronic infections are established. Chronic infection sets up a condition where microbial antigens induce a host inflammatory response and disrupt the innate lung defense. These aspects are thought to play a key role in the irreversible airway damage from which some patients ultimately die [1]–[4].

*Haemophilus influenzae* is a human-restricted respiratory pathogen that can either be capsulated (typable) or unencapsulated (nontypable). Encapsulated strains generally cause systemic infections, e.g., bacteremia and meningitis, whereas nontypable *H. influenzae* (NTHi) strains often cause non-invasive mucosal infections, e.g., otitis media, sinusitis, and conjunctivitis. Importantly, NTHi is encountered in a significant proportion in the lower airways of chronic patients suffering COPD, bronchiectasis or CF [5]. Studies addressing the hallmarks of *H. influenzae* infection in adult patients with obstructive lung diseases have been focused on COPD, where infection by NTHi is regarded as a marker of severe obstructive airflow and progression of the disease, and has been associated with decline in lung function and mortality [2]. Screening of COPD patients for *H. influenzae* during both scheduled and exacerbative assessments revealed persistent colonization with identical NTHi strains [6]. Genetic features associated with persistent NTHi strains are: (i) changes in the sequence of the outer membrane protein encoding *ompP2* gene over time [7], [8]; (ii) phase variation in the *hmw1A* and *hmw2A* genes, leading into a decreased expression of HMW1A and HMW2A adhesins over time [9]; (iii) adaptation in COPD of a clonally related group of NTHi strains with two IgA1 protease genes (*iga* and *igaB*) [10]. Conversely, acquisition of a new NTHi strain has been shown to be an important cause of lower respiratory tract infection associated with the occurrence of COPD exacerbations [11]. Newly acquired strains, isolated for the first time at exacerbation symptom onset, seem to mediate more neutrophil recruitment, adhere to epithelial cells in higher numbers, and induce more interleukin-8 (IL-8) release after interaction with airway epithelial cells than colonizers, isolated when the patient had no clinical signs of exacerbation [12]. Evidence also indicates that adults with COPD make new antibodies to strain-specific, surface-exposed epitopes on *H. influenzae* after exacerbations, and that the strain specificity of the immune response may favor further exacerbation by new strains [13]. Also, new strain exacerbations are associated with significantly greater increases from baseline in sputum TNF-α, neutrophil elastase and serum C-reactive protein than preexisting strain exacerbations [14].

Altogether, existing evidence suggests that persistent NTHi isolates may display differential features. In this study, we question whether series of persistent NTHi isolates with identical PFGE profile, recovered from consecutive acute exacerbations from three adults with a chronic lung disease, patients 1, 2 and 3, present distinctive genotypic or phenotypic features that could relate to persistence. These three retrospective series of NTHi isolates were tested in terms of cultured human respiratory epithelial infection, self-aggregation, biofilm formation and resistance to antimicrobial peptides (AMPs). Results were compared with those obtained on a panel of newly acquired isolates causing acute exacerbations. Comparison was made between isolates overall, between persistent and newly acquired strains, and between sequential clonal isolates within a patient. We further characterized the series of three isolates recovered from patient 1 in terms of growth rate, natural transformation frequency and mouse pulmonary infection. Finally, we genome sequenced this series of three clonal isolates collected from patient 1, which revealed a range of nonsynonymous single-nucleotide variants (SNVs) potentially linked to the observed phenotypes. Overall, we present a number of phenotypic and genomic traits that could be relevant for NTHi persistent infection.

## Methods

### Ethics Statement

This work was approved by the ‘Comité Etic d’Investigació Clínica, Hospital Universitari Bellvitge (HUB, Spain). A written or oral informed consent was considered not necessary because the source of bacterial isolates was anonymous and the study was retrospective. The institutional review board waived the need for written informed consent.

### Bacterial Isolates

From January 1997 to January 2003, a collection of NTHi isolates obtained from sputum samples from adult respiratory patients seen at the HUB was cryopreserved at −80°C. Sputa were collected during acute exacerbation, defined as any sustained increase in respiratory symptomatology compared with the baseline situation and requiring an increase in regular medication and hospital treatment. NTHi isolates were identified by conventional methodology and by mass spectrometry using a MALDI-Biotyper version 3.0 (Bruker), following the manufacturer’s recommendations. Serotyping was achieved with the latex agglutination Phadebact Haemophilus Test (Bactus AB). For this study, a retrospective review of computerized microbiological and medical charts was performed in patients seen at HUB during this period suffering a chronic lung disease, and with two or more cryopreserved NTHi isolates. Only NTHi isolates from good quality sputum samples (<10 squamous cells and >25 leukocytes per low-power field) and with a predominance of Gram negative cocobacillary forms were considered [15]. We selected 18 NTHi isolates from 6 patients with chronic lung disease and with 2 or more episodes of acute exacerbations due to NTHi (Table 1).

**Table 1:** Clinical and demographic characteristics of patients, antibiotic resistance patterns, PFGE profile and genetic features of NTHi isolates used in this study.

Patient	Gender	Age (years)	Disease	NTHi isolate (N°)	Isolation date	Antibiotic resistance	PFGE	f *lic2BC* presence	g *lic1A* (CAAT)_n_ (phase ON/OFF)
**1**	male	56	Bronchiectasis (BE)	1a (411)	17/2/1997	aAp, bTxS	A	−	17 (off)
				1b (584)	7/11/1997	Ap, TxS	A	−	17 (off)
				1c (1104)	9/11/1998	Ap, TxS	A	−	17 (off)
**2**	male	69	cCOPD	2a (628)	15/12/1997	Ap, TxS	B	+	17 (off)
				2b (920)	8/5/1998	Ap, TxS	B	−	17 (off)
**3**	female	73	Cystic fibrosis. BE	3a (735)	10/2/1998	dSusceptible	C	+	13 (on)
				3b (1340)	15/6/1999	Ap, TxS	D	+	14 (on)
				3c (1684)	3/10/2000	Ap, TxS	D	+	14 (on)
**4**	male	64	COPD. Emphysema	4a (629)	12/12/1997	TxS	E	+	28 (on)
				4b (846)	1/4/1998	TxS	F	+	6 (on)
				4c (2302)	8/3/2002	Susceptible	G	+	41 (on)
				4d (2612)	13/1/2003	Susceptible	H	+	25 (on)
**5**	female	68	COPD	5a (322)	12/12/1996	TxS	I	−	21 (on)
				5b (865)	14/4/1998	Susceptible	J	+	eND
				5c (1244)	8/3/1999	TxS	K	−	36 (on)
				5d (1715)	16/11/2000	Susceptible	L	−	27 (on)
**6**	male	38	COPD	6a (995)	30/6/1998	Susceptible	M	−	10 (on)
				6b (2116)	11/12/2001	Ap, TxS	N	−	ND

a Ap: ampicillin.

b TxS: Trimethoprim-sulfomethoxazole.

c COPD: Chronic Obstructive Pulmonary Disease.

d Susceptible: susceptible to all antimicrobials tested.

e ND: not determined.

f *lic2BC*: the *lic2BC* genes encode two glycosyltransferases involved in sugar extension from HepII in the LOS molecule.

g *lic1A*: the *lic1A* gene encodes a choline kinase. *lic1A* gene potential phase ON/OFF level of gene expression, based on the three translation initiation codons and two initiation codon-containing frames predicted for *lic1A* [46].

### Pulse Field Gel Electrophoresis (PFGE) and Molecular Typing

Strain relatedness was determined by PFGE. PFGE typing was performed on bacterial suspensions of *H. influenzae* grown on chocolate agar plates, as previously described [16], [17], with some modifications [18]. Briefly, bacterial suspensions were prepared in PIV (10 mM Tris-HCl [pH 8], 1 M NaCl) and adjusted to the same final concentration. The bacterial suspension was mixed with an equal volume of melted 1.5% low-melting point agarose (Life Technologies), to prepare DNA-agarose plugs with a volume of 20 µl each. These were incubated for 5 h at 37°C in 1 ml of ST buffer (6 mM Tris-HCl [pH 8]; 1 M NaCl; 0.1 M EDTA [pH 8]) containing 0.5% Brij-58, 100 mg/mL lysozyme and 50 mg/ml RNAse. The agarose plugs were transferred into ES buffer (1 M EDTA, 1% sarcosyl) with 1 mg/mL proteinase K (Sigma) and incubated for 16 h at 50°C. The plugs were rinsed three times at room temperature with TE buffer (10 mM Tris-HCl [pH 8]; 1 mM EDTA [pH 8]). The DNA-embedded plugs were digested with 5 U of *Sma*I (New England BioLabs) for 18 h at 25°C. DNA fragments were then separated in a 1% agarose gel (Megabase, BioRad) with 0.5% TBE buffer (45 mM Tris-base, 45 mM boric acid, 1.0 mM EDTA pH 8.0) in a contour-clamped homogenous electric field system (CHEF DR III; BioRad). Gels were run for 19 h at 14°C, using a constant voltage of 6 V/cm with an angle of 120° and an increasing pulse time from 1 s to 30 s. A bacteriophage λ low-range PFG marker (New England BioLabs) was used as a size standard. PFGE band patterns were analysed using the Fingerprinting II Software 3.0 (BioRad). Each cluster received a PFGE profile, named with a capital letter (Table 1). Persistent isolates were defined as those recovered in consecutive episodes from the same patient showing an identical PFGE pattern. A new acquired isolate was considered when its PFGE pattern was different to that of the strain isolated in the previous episode suffered by the same patient [19].

When required, chromosomal DNA was isolated. The *lgtF*, *lic2A*, *lic3A*, *lic3B* and *ompP5* genes were PCR-amplified using primers previously described [17]. For *lpsA*, primers lpsa-F1 (5′-TACTTAGCTCGCATTCTCAGGGCAGGGTGA) and lpsa-R1 (5′-AACACTCAAATGCTCATCAGAACAGGCTGA) were used. For *lic1A*, primers lic1A-F1 (5′-TCCACTATTAGGCGAAACCTTAGCTTC) and lic1A-R1 (5′-TAAATAACGAGTCAGTTTTACGCCACTT) were used. For *hmw1A*, primers hmw1A-7D (5′-CACTCGCTAGTGGTTGTTAATGATGAA) and hmw1A-rec-R1 (5′-GATTCGCAGAGATAGATTGACCTTTTG) were used. For *sapC*, primers sapC-F1 (5′-ATGCAAAATAAAGAACCTGATGAA), sapC-F2 (5′-GTTTATGGAATGCAATGTTTGCTA), sapC-R1 (5′-CTATTCTTGATGTTGATTGATGGC) and sapC-R2 (5′-ATACCTTTCAGTAGTCCTGCAATA) were used. For *kdkA*, primers kdkA-F1 (5′-ATGCACCAATTCCAACAAGATAAC) and kdkA-R1 (5′-TTATTGATGATAAGCTGACGTTAA) were used. For *lic1A*, *ompP5*, *sapC* and *kdkA*, PCR products were Sanger sequenced.

### Colony Immunoblot

PCho level was assessed as described previously, using mouse anti-PCho TEPC-15 antibody (Sigma) [20].

### Antimicrobial Susceptibility Testing

Antimicrobial susceptibility was determined by microdilution according to the criteria of the Clinical Laboratory Standards Institute (CLSI) [21], [22]. Ampicillin, amoxicillin-/clavulanic acid, cefuroxime, cefotaxime, ceftriaxone, imipenem, meropenem, tetracycline, chloramphenicol, trimethoprim/sulfamethoxazole, azithromycin, ciprofloxacin, levofloxacin and rifampin were tested. β-lactamase production was screened using the chromogenic cephalosporin method (nitrocefin disks, BD).

### Growth analysis

Growth was measured by using a BioScreen C instrument (BioScreen Instruments Pvt. Ltd.). Nine to twelve colonies of each strain were dispersed into 2 ml of sBHI medium, and 200 µl was added to wells of a BioScreen 100-well plate. Wells along the edges of each plate contained sBHI medium alone, as these wells were previously found to have anomalously slow growth, likely due to a lower temperature at the edges. Plates were incubated in the BioScreen plate at 37°C with agitation, and the OD_600_ was measured every 10 min for 24 h. The growth curve of each well was baseline corrected to its minimum value (these values were always within the range of values of control wells), and the average OD_600_ of the replicates at each time point was calculated.

### Transformation assays

*H. influenzae* cells were made competent by the transfer of sBHI medium-grown cells into MIV medium at an OD_600_ of 0.3 and incubation for 100 min at 37°C, as described previously [23]. These cells were incubated with 1 µg of MAP7 chromosomal DNA [23] per 1 ml of culture for 15 min at 37°C. Cells were then diluted and plated onto sBHI agar with and without novobiocin or nalidixic acid. Transformation frequencies were calculated as the number of novobiocin-resistant (Nov^r^), nalidixic acid-resistant (Nal^R^) transformants per cell.

### Antimicrobial Peptide Susceptibility Assay

Antimicrobial peptide susceptibility was assessed as described previously [24], adapted for *H. influenzae*. Bacteria grown on 10 ml sBHI were harvested (3500×r.p.m, 20 min, 20°C) in exponential growth (OD_650_ = 0.3). Bacteria were washed once with PBS and a suspension containing 10^5^ c.f.u./ml was prepared in 10 mM PBS (pH 6.5), 1% tryptone soy broth (Oxoid) and 100 mM NaCl. Aliquots (5 µl) of this suspension were mixed in 1.5 ml microcentrifuge tubes with various concentrations of polymyxin B (PxB) (prepared in 10 mM PBS (pH 6.5), 1% TSB and 100 mM NaCl). In all cases, the final volume was 30 µl. After 30 or 60 min of incubation at 37°C, the content of the tubes was plated on sBHI-agar. Alternatively, 5 µl of the bacterial suspensions were mixed with 25 µl of various concentrations of human β-defensin 1 (prepared in 10 mM PBS (pH 6.5), 1% TSB and 100 mM NaCl), incubated 1 h at 37°C, and plated on sBHI-agar. Colony counts were determined. Results are expressed as percentage of the colony count of bacteria not exposed to the antibacterial agent. Experiments were performed with duplicate samples on at least three independent occasions (n≥6). Mean±SD were calculated, and statistical comparison of means was performed using the two-tail *t* test (Prisma4 for PC). A p<0.05 value was considered statistically significant.

### Lipid A Purification and Analysis

Lipid A was extracted using an ammonium hydroxide/isobutyric acid method and subjected to negative-ion matrix-assisted laser desorption ionization time-of-flight (MALDI-TOF) mass spectrometry analysis [25]. NTHi isolates were grown on chocolate-agar plates; when required, strains were grown on sBHI-agar plates in the presence of a sub-lethal concentration of PxB (0.5 USP units/ml). Freshly grown bacteria were scraped from the plates, resuspended in 400 µl of isobutyric acid–1 M ammonium hydroxide (5∶3 [vol/vol]) and incubated in a screw-cap test tube at 100°C for 2 h, with occasional vortexing. Samples were cooled in ice water and centrifuged (2,000×g for 15 min). The supernatant was transferred to a new tube, diluted with an equal volume of water, and lyophilized. The sample was then washed twice with 400 µl of methanol and centrifuged (2,000×g for 15 min). The insoluble lipid A was solubilized in 100 to 200 µl of chloroform-methanol-water (3∶1.5∶0.25 [vol/vol/vol]). Analyses were performed on a Bruker Autoflex II MALDI-TOF mass spectrometer (Bruker Daltonics, Inc.) in negative reflective mode with delayed extraction. The ion-accelerating voltage was set at 20 kV. To analyze the samples, 1 to 3 µl of lipid A suspension (1 mg/ml) were desalted with a few grains of ion-exchange resin (Dowex 50W-X8; H) in a 1.5 ml microcentrifuge tube. A 1 µl aliquot of the suspension (50 to 100 µl) was deposited on the target and covered with the same amount of dihydroxybenzoic acid matrix (Sigma) dissolved in 0.1 M citric acid. Different ratios between the samples and dihydroxybenzoic acid were used when necessary. Alternatively, lipid A was mixed with 5-chloro-2-mercapto-benzothiazole (Sigma) at 20 mg/ml in chloroform-methanol (1∶1 [vol/vol]) at a ratio of 1∶5. Each spectrum was an average of 300 shots. A peptide calibration standard (Bruker Daltonics) was used to calibrate the MALDI-TOF. Further calibration for lipid A analysis was performed externally using lipid A extracted from *E. coli* strain MG1655. Interpretation of the negative-ion spectra is based on earlier studies showing that ions with masses higher than 1,000 gave signals proportional to the corresponding lipid A species present in the preparation [26]–[29].

### Bacterial Aggregation Assay

Three or four colonies of NTHi or *E. coli* grown on chocolate-agar or LB-agar plates, respectively, were inoculated into 20 ml sBHI (NTHi) or LB (*E. coli*) media, grown overnight, diluted in the same medium to OD_600 nm_ = 1, and left standing at room temperature for 7 h (starting volume ∼20 ml). The viability of each culture was tested by serial dilution and plating on sBHI-agar or LB-agar at the beginning of each experiment (t = 0). OD_600 nm_ readings were performed at regular time intervals on 500 µl aliquots collected from the top of each bacterial suspension. At least four independent experiments (n≥4) were performed for each strain. Mean±SD were calculated, and statistical comparison of means was performed using the one-way ANOVA followed by the Fisher’s Protected Least Significant Differences (PLSD) tests (StatViewGraphics 5.0 for Windows (SAS Institute Inc^©^). A p<0.05 value was considered statistically significant.

### Biofilm Formation

Biofilm formation under flow conditions was assessed using 60 ml microfermenters (Pasteur Institute, Laboratory of Fermentation) with a continuous flow of medium (40 ml/h) and constant aeration with sterile compressed air (0.3 bar). Submerged Pyrex glass slides served as the growth substratum. Three or four colonies of each strain grown on chocolate-agar plates were inoculated in 20 ml sBHI medium and incubated under shaking up to OD_600 nm_ = 1. ∼10^8^ bacteria from this culture were used to inoculate the microfermenter, which was run at 37°C for 16 h. The viability of each inoculated bacterial aliquot was tested by serial dilution and plating on sBHI-agar. To quantify the biofilm formed, bacteria adhered to the Pyrex slides were resuspended in 10 ml PBS. The OD_600 nm_ of the suspensions was determined. Experiments were performed on at least three independent occasions (n≥3). Mean±SD were calculated, and statistical comparison of means was performed using the two-tail *t* test (Prisma4 for PC). A p<0.05 value was considered statistically significant.

### Cell Culture and Bacterial Infection

A549 human immortalised type II pneumocytes, ATCC CCL-185, were maintained as previously described [30]. Cells were seeded to 6×10^4^ cells/well in 24-well tissue culture plates for 32 h and serum-starved for 16 h before infection by replacement of medium lacking FBS. A confluence of 90% was reached at the time of infection. When necessary, cells were seeded for a longer period of time to reach an over-confluent monolayer (approximately 8×10^5^–1×10^6^ cells/well). Stationary phase grown NTHi were recovered with 1 ml PBS from a chocolate-agar plate and adjusted to OD_600_ = 1 (10^9 ^c.f.u./ml). Cells were infected in 1 ml EBSS (Earle’s Balance Salt Solution) with the freshly obtained bacterial suspension, at approximately MOI 100∶1, for 30 min, washed five times with PBS, and lysed with 300 µl of PBS-Saponin 0.025% for 10 min at room temperature. Serial dilutions were plated on sBHI-agar and adhesion scored as c.f.u./well. Experiments were performed in triplicate on at least three independent occasions (n≥9). Mean±SD were calculated, and statistical comparison of means was performed using the two-tail *t* test (Prisma4 for PC). A p<0.05 value was considered statistically significant.

### Secretion of IL-8

A549 cells were maintained and infected for 2 h, as described above. Cells were washed 3 times with PBS, and incubated with fresh medium containing gentamicin (100 µg/ml) for 6 or 20 h. Supernatants were removed from the wells, cell debris was removed by centrifugation, and samples were frozen at −80°C. IL-8 levels in the supernatants were measured using an ELISA kit (Innova). Infection experiments were carried out in duplicate and on at least two independent occasions (n≥4). Mean±SD were calculated, and statistical comparison of means was performed using the two-tail *t* test (Prisma4 for PC). A p<0.05 value was considered statistically significant.

### Mouse Assays

A mouse model of NTHi pulmonary infection was used, as described previously [31]. CD1 female mice (4 to 5 weeks old) were purchased from Charles River Laboratories (France) and housed under pathogen-free conditions at the Institute of Agrobiotechnology of the Universidad Pública de Navarra (UPNA) facilities (registration number ES/31-2016-000002-CR-SU-US). Animal handling and procedures were in accordance with the current European (Directive 86/609/EEC) and National (Real Decreto 53/2013) legislations, following the FELASA and ARRIVE guidelines, and with the approval of the UPNA Animal Experimentation Committee (Comité de Ética, Experimentación Animal y Bioseguridad-CEEAB, http://www.unavarra.es/invest/comiteEtica.htm) and the local Government authorization (approval reference number PI-010-2012). For NTHi infection, bacteria were recovered with 1 ml PBS from a chocolate-agar plate grown for 16 h, to obtain a suspension of ∼5×10^9^ c.f.u./ml. Before infection, mice were anesthetized by intraperitoneal inoculation of a mixture of ketamine-xylazine (3∶1). Each mouse received 20 µl of inoculum (∼10^8^ c.f.u.) intranasally. Groups of 5 mice were euthanized and necropsied at selected intervals. Lungs were aseptically removed, individually weighed in sterile bags (Stomacher80, Seward Medical), homogenized, and serially ten-fold diluted in PBS. Each dilution was spread on sBHI-agar plates to determine the number of viable bacteria (detection limit <10 c.f.u./lung). The individual number of c.f.u./lung was logarithmically transformed for data normalization. Statistical comparisons of mean log_10_ c.f.u./lung were performed by a one-way ANOVA followed by the Fisher’s Protected Least Significant Differences (PLSD) tests using the Stat View Graphics 5.0 for Windows (SAS Institute Inc) statistical package. A p<0.05 value was considered statistically significant.

### Sequencing, Assembly and Comparison of three Clonal Isolates’ Genomes

Genomic DNA samples were extracted from the three isolates from patient 1, NTHi1a, 1b, 1c, and a derivative of the completely sequenced isolate 86-028NP, RR3131 [32], [33] using standard methods [23]. Samples were converted to sequencing libraries using the Nextera-XT kit (Illumina) and included in a multiplexed Illumina HiSeq RapidRun, collecting 2×101 nucleotide paired-end reads. Raw data was then converted to FastQ format using bcl2fastq-v1.8.4 (Illumina). Genome assemblies were performed with a 5-step pipeline: (i) Nextera adaptor sequences were trimmed from reads using Trimmomatic-v0.30 [34]; (ii) overlapping paired-end reads were joined using COPE-v1.1.2 [35]; (iii) error correction was performed using ALLPATHS-LG stand-alone ErrorCorrectReads.pl utility [36]; (iv) optimal kmer size was estimated using kmergenie-v1.5658 [37]; and (v) finally, *de novo* assembly was performed using Ray-v2.2.0 [38]. Assembly quality was assessed using whole-genome alignment with Mauve-v2.4 [39] of the contigs generated from the RR3131 strain to the 86-028NP complete genome sequence from NCBI (GenBank accession NC_007146.2). Synteny was evaluated iteratively applying the Mauve “Move Contigs” utility with 86-028NP as the reference sequence. Finally, contigs were annotated using the automated web service RAST [40], and the resulting GFF3 output was parsed to generate a BED file of protein coding sequences. Sequence data for strains NTHi1a, 1b and 1c were deposited to the NCBI under project accession number PRJNA240637.

To identify variants between the three isolates, the original paired-end reads of each sample were realigned to each set of contigs using the mem algorithm in bwa-v0.7.5a-r405 [41], and samtools/bcftools were used to identify single-nucleotide variants and small indels distinguishing the three strains [42]. Variant calls in VCF format were then filtered to exclude those positions with “heterozygous” or ambiguous base calls in any of the three strains, as these likely represent systematic sequencing artifacts. The remaining variants were then intersected against the BED annotation using the intersect utility in bedtools-v2.16.2 [43] to obtain a list of variants within coding sequences. The bedtools utility getFasta was used to extract the relevant ORFs, and the R package seqinr [44] was used to perform translations of variant coding sequences. Web-based BLASTP of the set of protein sequences containing nonsynonymous differences was used to manually curate the RAST annotations and facilitate literature searches for previous experimental work in *H. influenzae*.

## Results

### NTHi Isolates Collected from Adult Patients with Obstructive Lung Disease

NTHi isolates used in this study were recovered from sputum samples from 18 episodes of acute exacerbations in 6 adult patients with chronic lung diseases (4 COPD, 1 bronchiectasis, 1 cystic fibrosis and bronchiectasis). Patients were attended at Hospital Universitario Bellvitge, Spain, when they had symptoms suggesting an exacerbation. Table 1 summarizes clinical and demographic characteristics of these patients, antibiotic resistance patterns, pulse field gel electrophoresis (PFGE) profiles, and genetic features of NTHi isolates.

NTHi isolates were typed by PFGE and divided in two groups (Table 1): (i) persistent isolates had identical PFGE profile to those isolated from previous sputa in the same patient. Patients 1, 2 and 3 belonged to this category, and rendered three series of clonal isolates that we considered as persistent. (ii) Newly acquired isolates showed a PFGE profile different than those isolated from previous sputa in the same patient. Patients 4, 5 and 6 belonged to this category, and isolates recovered from these patients were considered as newly acquired. The series recovered from patient 3 contained one newly acquired (NTHi3a) and two persistent isolates (NTHi3b and 3c).

Antibiotic susceptibility of the 18 NTHi isolates is shown in Table 2. All isolates were susceptible to amoxicillin/clavulanic acid, cefuroxime, ceftriaxone, cefotaxime, imipenen, meropenem, tetracycline, chloramphenicol, trimethoprim/ sulfamethoxazole, azithromycin, ciprofloxacin, levofloxacin and rifampicin. Conversely, three isolates were resistant to ampicillin due to β-lactamase production, and five isolates presented intermediate resistance without β-lactamase production. All but six isolates were resistant to trimethoprim/ sulfamethoxazole. Finally, persistent isolates were identical in terms of antibiotic susceptibility, within each patient.

**Table 2:** Minimal inhibitory concentrations (µg/ml) of 14 antimicrobials against 18 NTHi isolates using the microdilution method according to CLSI breakpoint criteria^a^.

Patient	NTHi isolate	AM	AMC	CFU	CTX	CRO	IMI	MER	TET	CHL	TxS	AZI	CIP	LEV	RIF
**1**	1a	2	4/2	2	0.25	≤0.12	0.25	0.5	≤1	≤1	≥4/76	0.5	0.03	≤0.5	≤0.25
	1b	2	4/2	2	0.25	≤0.12	0.25	0.5	≤1	≤1	≥4/76	0.5	0.03	≤0.5	≤0.25
	1c	2	4/2	2	0.25	≤0.12	0.25	0.5	≤1	≤1	≥4/76	0.5	0.03	≤0.5	≤0.25
**2**	2a	2	2/1	1	0.25	≤0.12	0.25	0.25	≤1	≤1	≥4/76	0.25	0.03	≤0.5	≤0.25
	2b	2	2/1	1	0.25	≤0.12	0.25	0.25	≤1	≤1	≥4/76	0.25	0.03	≤0.5	≤0.25
**3**	3a	0.5	1/0.5	2	0.06	≤0.12	0.5	0.25	≤1	≤1	0.5/9.5	0.5	0.03	≤0.5	≤0.25
	3b	≥ 8^b^	1/0.5	0.5	0.06	≤0.12	0.25	0.25	≤1	≤1	≥4/76	1	0.03	≤0.5	≤0.25
	3c	≥ 8^b^	1/0.5	0.5	0.06	≤0.12	0.25	0.25	≤1	≤1	≥4/76	1	0.03	≤0.5	≤0.25
**4**	4a	0.25	0.5/0.25	1	0.06	≤0.12	0.25	0.25	≤1	≤1	≥4/76	0.12	0.03	≤0.5	≤0.25
	4b	1	2/1	2	0.12	≤0.12	1	0.25	≤1	≤1	≥4/76	1	0.03	≤0.5	≤0.25
	4c	0.25	0.5/0.25	1	0.06	≤0.12	0.12	0.25	≤1	≤1	0.5/9.5	0.5	0.03	≤0.5	≤0.25
	4d	0.25	0.5/0.25	1	0.06	≤0.12	0.5	0.25	≤1	≤1	0.5/9.5	0.5	0.03	≤0.5	≤0.25
**5**	5a	0.25	0.5/0.25	1	0.06	≤0.12	0.25	0.25	≤1	≤1	≥4/76	0.12	0.03	≤0.5	≤0.25
	5b	1	1/0.5	2	0.06	≤0.12	0.5	0.25	≤1	≤1	0.5/9.5	0.5	0.03	≤0.5	≤0.25
	5c	0.25	0.5/0.25	1	0.06	≤0.12	1	0.25	≤1	≤1	≥4/76	0.12	0.03	≤0.5	≤0.25
	5d	0.5	1/0.5	2	0.06	≤0.12	0.12	0.25	≤1	≤1	0.5/9.5	2	0.12	≤0.5	≤0.25
**6**	6a	0.25	0.5/0.25	1	0.06	≤0.12	0.12	0.25	≤1	≤1	≥4/76	0.12	0.03	≤0.5	≤0.25
	6b	≥ 8^b^	0.5/0.25	1	0.06	≤0.12	0.12	0.25	≤1	≤1	≥4/76	1	0.03	≤0.5	≤0.25

a CLSI: Clinical and Laboratory Standard Institute. Breakpoint Criteria (S, susceptible; I intermediate; R, resistant): AM (Ampicillin; S≤1, I = 2, R≥4); AMC (Amoxicillin-/clavulanic acid; S≤4/2, R≥8/4); CFU (Cefuroxime; S≤4, I = 8, R≥16); CTX (Cefotaxime; S≤2); CRO (ceftriaxone; S≤2); IMI (Imipenem; S≤4); MER (Meropenem; S≤0.5); TET (Tetracycline; S≤2, I = 4, R≥8); CHL (Chloramphenicol; S≤2, I = 4, R≥8); TxS (Trimethoprim/sulfamethoxazole; S≤0.5/9.5, I = 1/19–2/38, R≥4/76); AZI (Azithromycin; S≤4); CIP (Ciprofloxacin; S≤1); LEV (Levofloxacin; S≤2); RIF (Rifampin; S≤1, I = 2, R≥4). Ref CLSI 2013.

b β-lactamase producing isolate.

### Lipooligosaccharide Gene Distribution and Phase Variation on NTHi Isolates Collected from Adult Patients with Obstructive Lung Disease

Lipooligosaccharide (LOS) is a heterogeneous surface structure on NTHi, partly due to a strain variable distribution of the genes encoding the enzymes required for its biosynthesis, being some of them phase variable [45]. We used PCR and DNA sequencing to evaluate all isolates for the presence of genes encoding enzymes required for the biosynthesis of LOS. Five of these genes were present in all strains: *lgtF* (glycosyltransferase responsible for adding glucose as the first sugar to HepI in the LOS molecule), *lic2A* (glycosyltransferase required to add a digalactose to the LOS), *lpsA* (glycosyltransferase responsible for adding glucose or galactose as the first sugar to HepIII in the LOS molecule), *lic3A* and *lic3B* (sialyltransferases). The presence of *lic2B* and *lic2C* was variable (Table 1). Given that *lic2B* and *lic2C* encode two glycosyltransferases involved in sugar extension from HepII in the LOS molecule, this extension is likely to be variable among strains. PCho addition to the LOS molecule requires the *lic1ABCD* operon [20], present in all strains tested. The *lic1A* gene is phase variable due to a variable number of tetranucleotide (5′-CAAT-3′) repeats in its coding sequence. Gel electrophoresis revealed *lic1A* size differences among isolates, correlating with *lic1A* gene sequences containing between 6 and 41 5′-CAAT-3′ repeats (Table 1). The *lic1A* gene has been shown to present 3 potential translational initiation codons and 2 potential initiation codon-containing frames [46]. Taking these alternatives into consideration, the *lic1A* gene was likely to be phase ON in most NTHi strains tested. Only strains with 17 repeats in the *lic1A* gene seemed to be phase OFF, independently of the translational initiation codon and frame considered (Table 1). Of note, when PCho decoration was monitored by colony immunoblot, it was found to be present in all strains. NTHi colonies were observed to exhibit variable reactivity with TEPC-15, classified as high and low, as previously reported [20]. The distribution of PCho-reactive colony types was variable among strains (Table 3).

**Table 3:** PCho expression by NTHi isolates.

Patient	Isolate	aHigh reactivity (% of colonies)	Low reactivity (% of colonies)
**1**	NTHi1a	71.1	28.9
	NTHi1b	51.2	48.8
	NTHi1c	77.5	22.5
**2**	NTHi2a	32.2	67.8
	NTHi2b	5.9	94.1
**3**	NTHi3a	93.8	6.2
	NTHi3b	0	100
	NTHi3c	13.8	86.2
**4**	NTHi4a	100	0
	NTHi4b	10.8	89.2
	NTHi4c	7.3	92.7
	NTHi4d	47.8	52.2
**5**	NTHi5a	100	0
	NTHi5b	96.5	3.5
	NTHi5c	94.3	5.7
	NTHi5d	3.8	96.2
**6**	NTHi6a	95.2	4.8
	NTHi6b	7.2	92.8

a PCho expression was detected by colony immunoblot with the anti-PCho monoclonal antibody TEPC-15. Strains were classified by colony reactivity with the TEPC-15 antibody as previously described [20]. The intensity of TEPC-15 signal rendered by each colony was determined as high or low. Percentages were calculated by assessing colony reactivity for at least 300 colonies per strain, in at least two independent experiments.

### Infection of Airway Epithelial Cells by NTHi Isolates Collected from Adult Obstructive Respiratory Patients

**Figure 1:**
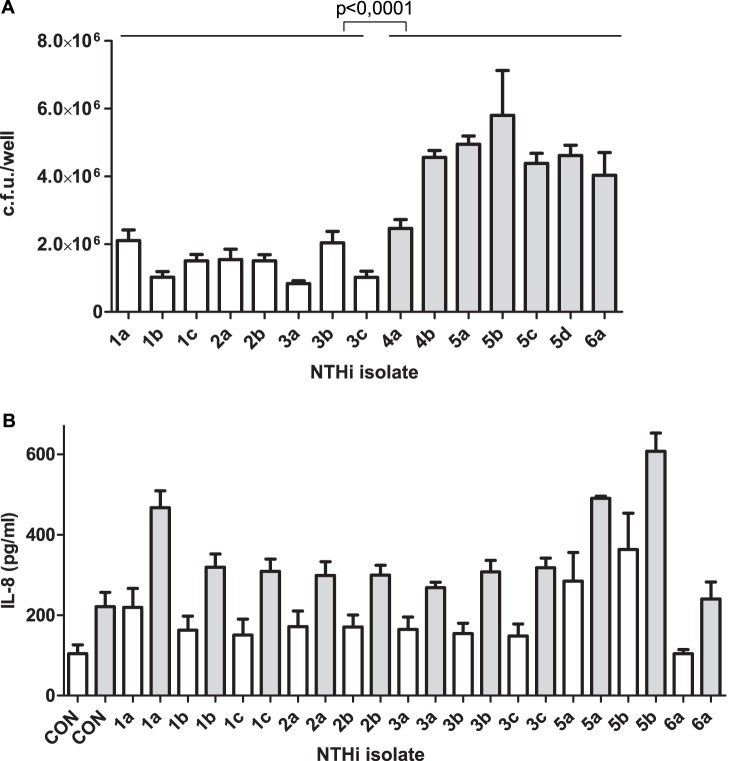
Infection of epithelial cells by NTHi isolates recovered from chronic respiratory patients. (**A**) NTHi adhesion to A549 epithelial cells. NTHi cells were incubated with A549 cells for 30 min. Bacterial adhesion was quantified by lysis, serial dilution and viable counting on sBHI-agar plates. Mean numbers for newly acquired strains (grey bar) were significantly higher (p<0.0001) than those obtained for strains recovered from patients 1, 2 and 3 (white bar). (**B**) Stimulation of IL-8 secretion by infection for NTHi clinical isolates. Quantification by ELISA of IL-8 secreted to the supernatant by A549 cells upon infection with NTHi1a, 1b, 1c, 2a, 2b, 3a, 3b, 3c, 5a, 5b, 5c 5d and 6a. [IL-8] was quantified at 6 (white bar) and 20 (grey bar) h post-infection.

Bacterial pathogens gaining access to the lower airways of patients are likely to infect airway epithelial cells, and epithelial adhesion is in many cases an important stage of the infectious process [47]. We observed that adhesion of persistent NTHi strains recovered from patients 1 to 3 to A549 airway epithelial cells was significantly lower than that shown by newly acquired strains from patients 4 to 6 (Fig. 1A). *hmw1A* and *hmw2A* gene expression has been shown to decrease for persistent NTHi over time [9]. PCR evaluation showed that the *hmw1A* gene is absent in NTHi strains recovered from patients 1 and 2, limiting its potential implication in the observed phenotype.

P5 has been shown to be a bacterial ligand for the carcinoembrionic antigen molecule-1 CEACAM1 receptor [48]. PCR amplification showed that the *ompP5* gene was size invariable among the isolates tested for epithelial adhesion. Sequence variability of the *ompP5* gene has been previously reported [17]. For this reason, we sequenced each predicted OmpP5 extracellular loop (a prediction based on a PRED-TMBB β-barrel) on the NTHi isolates under study. The *ompP5* sequence was heterogeneous among strains; however, clonal isolates showed identical sequence in loops 1 to 5 (Table S1 in File S1). A comparable motif distribution for loop 1, encompassing GINNNGAIK/Q (red) or GLRALARE (blue) sequence, has been reported previously [49]. Loop sequences highlighted in grey were found in our previous study [17].

NTHi infection triggers the secretion of pro-inflammatory cytokines by epithelial cells [50]. A549 cell infection by strains NTHi1a, 5a and 5b promoted the secretion of IL-8 levels significantly higher than the basal level registered for non-infected cells, both at 6 and 20 h post-infection (Fig. 1B). Most other strains promoted the secretion of slight but not significant IL-8 levels; NTHi6a did not stimulate IL-8 secretion above the level seen in non-infected cells.

Collectively, these results showed that persistent NTHi isolates have low rates of adhesion to cultured epithelial cells, compared to the newly acquired strains tested. This observation was not associated with the variable sequence of the *ompP5* gene. In general, these isolates induced relatively low levels of epithelial IL-8 secretion.

### Self-aggregation and Biofilm Formation by Persistent NTHi Isolates

**Figure 2:**
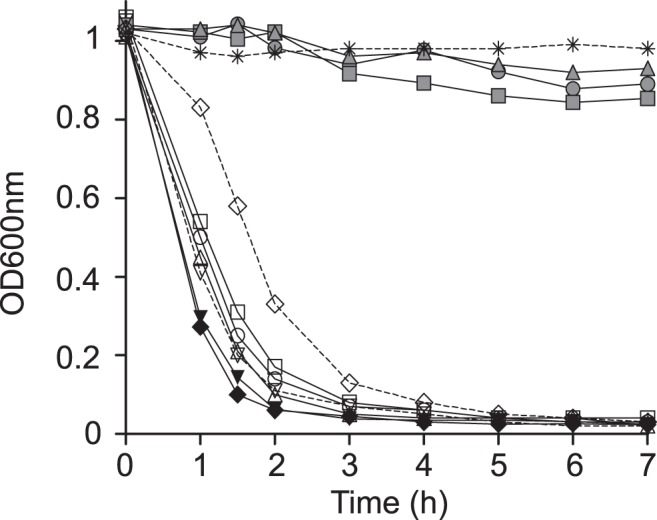
*H. influenzae* clinical isolates self-aggregation. Tube-settling experiment of stationary phase cultures of isolates NTHi1a (open circles), NTHi1b (open squares), NTHi1c (open triangles), NTHi2a (black inverted triangle), NTHi2b (black diamond), NTHi3a (grey circle), NTHi3b (grey square), NTHi3c (grey triangle), NTHi5b (open inverted triangle) and NTHi6a (open diamond), after incubation at room temperature for 7 h. *E. coli* C600 (asterisk) was used as a negative control. Bacterial aggregation was quantified by measuring the decrease of absorbance at OD600nm. NTHi1a-1c self-aggregated significantly slower than NTHi2a-2b (p<0.0001 at 1 h; p<0.001 at 1.5 h). NTHi6a aggregated slower than strains from patients 1 and 2 (p<0.0001 at 1 and 1.5 h), and faster than isolates from patient 3 (p<0.0001, all time points tested).

NTHi self-aggregates, which may promote microcolony formation on host cell surfaces [51]. We asked whether self-aggregation differs between the NTHi isolates under study, by using tube-settling assays and monitoring the optical density of bacterial suspensions over time with a non-aggregating *E. coli* strain C600 as control. Strains recovered from the same patient self-aggregated at the same rate. The series isolated from patient 3 did not self-aggregate (p<0.0001 from 1 h to the end of the assay). The series isolated from patient 1 self-aggregated significantly slower than that recovered from patient 2 at early time points (p<0.0001 at 1 h; p<0.001 at 1.5 h). NTHi6a presented OD_600nm_ values higher than those obtained for strains from patients 1 and 2 (p<0.0001 at 1 and 1.5 h), and lower than isolates from patient 3 (p<0.0001, all time points tested) (Fig. 2, Table S2 in File S1). We have previously reported that bacteria lacking PCho self-aggregate slightly faster [31]. All strains tested displayed PCho, excluding its association with the observed phenotypes.

We also asked whether the available persistent isolates could form biofilms. We tested the formation of biofilm communities under continuous-flow culture conditions in microfermenters by measuring the turbidity of bacterial suspensions detached from removable glass slides. No biofilm could be visualised among isolates from patients 1, 2 and 3 (Table 4). NTHi5a, 5b and 6a were tested as a sample of newly acquired strains, and formed biofilms. NTHi biofilm growth has also been related to PCho [31]. Given that all strains tested for biofilm growth displayed PCho, we excluded an association between PCho and the observed phenotypes. In sum, we observed that, under the conditions tested, persistent NTHi isolates recovered from patients 1 to 3 do not develop biofilm communities.

**Table 4:** Biofilm formation under continuous-flow conditions, in microfermenters containing glass slides where bacteria formed the biofilm.

Patient	Isolate	OD_600nm_ of bacterial suspension detached from glass slide
**1**	NTHi1a	0.001
	NTHi1b	0.001±0.001
	NTHi1c	0.004±0.001
**2**	NTHi2a	0.0005±0.0007
	NTHi2b	0.0015±0.002
**3**	NTHi3a	0.0035±0.001
	NTHi3b	0.012±0.016
	NTHi3c	0.154±0.21
**5**	NTHi5a	1.31±0.28^a^
	NTHi5b	1.65±0.28^b^
**6**	NTHi6a	1.57±0.35^a^

Data shown are means and SD of absorbance values, compared by a two tail *t* test. NTHi5a and NTHi6a formed a biofilm significantly higher than the non-existing ones obtained for isolates from patients 1, 2 and 3, ^a^p<0.005. NTHi5b formed a biofilm significantly higher than the non-existing ones obtained for isolates from patients 1, 2 and 3, ^b^p<0.0005.

### Persistent NTHi Isolates Display Increasing Resistance to Antimicrobial Peptides

Antimicrobial peptides (AMPs) are positively charged innate immunity soluble peptides whose action is initiated through electrostatic interaction with the bacterial surface, the anionic lipid A moiety of the lipopolysaccharide (LPS) in Gram-negative bacteria [52]–[55]. Polymyxins are pentacationic amphipathic lipopeptide antibiotics active against Gram-negative bacteria and, similar to AMPs, they interact with the anionic LPS [56], [57]. We examined the susceptibility of the three series of clonal NTHi isolates to AMP mediated killing by incubating them for 60 min with a range of concentrations of polymyxin B (PxB). Resistance to killing varied modestly, but the later persistent isolates were consistently more resistant than the corresponding original isolates (Table 5). This observation became clearer when strains were incubated with PxB for 30 min (Table 6). Likewise, we found increasing resistance to the natural AMP human β-defensin-1 (hBD-1) among isolates with the same PFGE profile (Table 6). In sum, a progressive increasing resistance to PxB and hBD-1 could be observed on the series of persistent NTHi isolates under study.

**Table 5:** NTHi strains resistance to Polymyxin B (µg/ml) mediated killing after a 60 min bacterial incubation with the AMP.

Patient	Isolate			Polymyxin B (µg/ml)			
		1	0.75	0.5	0.25	0.125	0
**1**	NTHi1a	1.6±1	5.4±3	90±2.1	100	100	100
	NTHi1b	3±1.1	11.1±1.3^a^	86.9±4.7	99.6±7.4	95.8±3.5	100
	NTHi1c	3±1.7	8.8±0.8	85.8±1.7	95.1±9.9	97.2±6	100
**2**	NTHi2a	1.4±0.9	6.7±2	86.1±10.1	95.6±13.5	100	100
	NTHi2b	7.1±2.1^a^	11.9±2.5^a^	80.6±6.4	94.8±1.2	99±8	100
**3**	NTHi3a	4±3	8.1±1.9	90.2±0,9	100	100	100
	NTHi3b	12±4.5	19.2±10.5	78.7±7.9	98.4±4.8	100	100
	NTHi3c	9±1.1	19.5±4.6	98.3±9.2	98.9±6.6	100	100
**4**	NTHi4a	3.9±0.9	10.7±2.8	83.2±3.2	100	98.8±2.5	100
**5**	NTHi5a	4.6±1.2	7±2.8	86.8±6.2	100	100	100
	NTHi5b	3.8±2.2	8.7±3.4	83.5±11.8	100	100	100
**6**	NTHi6a	8.8±3.7	16±5.9	86.8±2.1	100	100	100

a p<0.05 vs. first strain with identical PFGE profile isolated from each patient.

**Table 6:** NTHi strains resistance to Polymyxin B and to human β-defensin 1 mediated killing after a 30 min bacterial incubation with the AMP.

AMP	Patient	Isolate	[AMP] (µg/ml)			
			1	0.75	0.5	0
**PxB**	**1**	NTHi1a	11.8±0.9	30.9±1.7	96±12.7	100
		NTHi1b	35.5±9.6^a^	69.3±9.4^a^	100	100
		NTHi1c	36.7±8^a^	78.1±9.2^a^	100	100
	**2**	NTHi2a	12.9±1.5	54.6±11.1	96.7±5.1	100
		NTHi2b	37.2±6.3^a^	74.3±2^a^	99.8±9.9	100
	**3**	NTHi3a	26.5±7.3	49±2.2	98.8±7.4	100
		NTHi3b	34.4±3.4	55.4±11.5	100	100
		NTHi3c	51.7±6.1^a^	75.1±4^a^	100	100
**hBD-1**	**1**	NTHi1a	57.3±3.8	88.8±7.7	ND*	100
		NTHi1b	84.5±3.7^a^	100±5	ND	100
		NTHi1c	91.2±4^a^	100	ND	100
	**2**	NTHi2a	60.7±7.5	91.7±3.7	ND	100
		NTHi2b	87.5±3.5^a^	100	ND	100
	**3**	NTHi3a	68.3±9.6	90.6±3.7	ND	100
		NTHi3b	87.1±0.6	100	ND	100
		NTHi3c	94.8±2.8^a^	100	ND	100

*ND = not determined.

a p<0.05 vs. first strain with identical PFGE profile isolated from each patient.

### Increasing Resistance to AMPs is not Related to Changes in LOS Decoration with PCho or Lipid A Acylation

NTHi resistance to AMPs has been related to LOS decoration with PCho [31], [58]. All strains tested for AMPs susceptibility displayed PCho, excluding its involvement in the observed increasing resistance. Lipid A acylation has also been linked to NTHi resistance to AMPs [59]. We characterized the lipid A moieties of strains NTHi1a to 1c, 2a, 2b, 3a to 3c, and 5a by MALDI-TOF mass spectrometry. Spectra were similar for the isolates tested (Figure S1A in File S1). Previous studies determined that the lipid A disaccharide backbone of NTHi is composed of two 2-amino-2-deoxyglucose residues (GlcNI and GlcNII) linked by a β-(1–6) glycosidic linkage and phosphorylated at positions 1 and 4′. The C2/C2′ and C3/C3′ positions are substituted by amide-linked and ester-linked 14∶0(3-OH), respectively. Fatty acid chains on C3′ and C2′ are further esterified by 14∶0 [60]. The isolates analyzed in this work showed a major lipid A species corresponding to the same hexa-acylated form (*m/z* 1,824) described by Mikhail and co-workers. Other ion peaks detected (*m/z* 1,744, and *m/z* 1,388) had also been previously found [60]. These peaks may represent the monophosphorylated hexa-acylated lipid A, and a tetra-acyl lipid A containing two 2-amino-2-deoxyglucose residues, two phosphates, three 14∶0(3-OH), and one 14∶0(3-OH), respectively (Figure S1A and Table S3 in File S1).

Together, the increasing resistance to PxB and to hBD-1 observed on the three series of persistent isolates could not be associated with variations in PCho decoration or in the lipid A acylation profile of the LOS molecule.

### Persistent Isolates from Patient 1 Display Similar Growth, Competence and Murine Pulmonary Infection Capacity

We further characterized one series of isolates identical by PFGE but distinct in terms of resistance to AMPs. We selected the three isolates recovered from patient 1, NTHi1a, 1b and 1c. We first characterized the lipid A moieties when bacteria were grown in the presence of sub-lethal concentrations of PxB. Results were identical to those obtained when bacteria were grown without PxB (Figure S1A in File S1). Next, we compared the growths of the three isolates in rich broth (sBHI medium) by using a BioScreen incubator. The three isolates had growth curves indistinguishable from each other (Figure S1B in File S1). Growth was compared with that of the reference strain *H. influenzae* RdKW20. The four strains tested rendered identical slopes. *H. influenzae* is naturally competent. If sequence homology permits, incoming DNA can recombine with the chromosome. When this recombination changes the genotype, the cell is said to be transformed. Evidence indicates that NTHi strains can differ considerably in the amount of DNA they take up and recombine [61]. Given that natural transformation is a major mechanism of genetic exchange that shapes bacterial genomes and allows pathogens to evade the host immune response, we asked if NTHi1a, 1b, 1c are competent and, if so, if their competence phenotypes differ. Competence was assessed by transformation assays. The three strains developed competence under the conditions tested, and displayed comparable transformation phenotypes (Figure S1C in File S1). Finally, pulmonary infection by NTHi1a, 1b and 1c was assessed by intranasal inoculation of CD1 mice, and quantification of bacterial loads from lung homogenates of infected mice was performed at 24 and 48 h post-infection (PI). At 24 h PI, the three strains delivered counts indistinguishable from each other. At 48 h PI, infections were almost cleared in the three groups of animals (Figure S1D in File S1).

In sum, the series of clonal isolates recovered from patient 1 showed comparable growth, transformation frequency and murine pulmonary infection rate. Together, these clonal isolates presented similar phenotypic traits, except for their resistance to AMPs.

### Genome Sequence Comparison of Serial Isolates NTHi1a, 1b, 1c Reveals Amino Acid Differences in the Permease SapC, four Heme uptake Systems, the Kdo Kinase KdkA, the Long-chain Fatty Acid Transporter OmpP1, and in the Phosphoribosylamine Glycine Ligase PurD

Given that the three isolates from patient 1 had the same PFGE profile and were phenotypically indistinguishable, except for their resistance to AMPs, increasing between NTHi1a and isolates NTHi1b and 1c, we hypothesized that the three isolates were likely drawn from the same population of *H. influenzae*, and that host selection pressures may have caused the change in AMP resistance. To confirm that the isolates were closely related, and to identify candidate genetic variants responsible for the phenotypic change, we performed whole genome sequencing on all three. As a control to evaluate the accuracy and completeness of our genome assembly pipeline, we included a derivative of the completely sequenced *H. influenzae* isolate 86-028NP (RR3131) [32], [33]. Comparison of the contigs generated for this control strain to the complete sequence of 86-028NP using the Mauve aligner [39] demonstrated that the sequencing and assembly was not only nearly complete (99% of the genome was covered, with most of the missing 20,000 bp accounted for by the unassembled rDNA repeats (Figure S2 in File S1), but also highly accurate (all 76 of the single-nucleotide variants detected between the contigs and the complete sequence were previously identified and no other variants were detected). The high quality of the RR3131 assembly gave us high confidence in the three bronchiectasis isolate draft assemblies. Summary statistics for the sequencing and assembly of all four sequenced isolates are provided in Table S4 in File S1.

Isolates NTHi1a, 1b and 1c were extremely closely related. Synteny was evaluated by iteratively reordering the contigs against the 86-028NP reference using Mauve; this found that gene order between the three isolates was highly conserved, corroborating the PFGE fingerprints but at higher resolution (Figure S3 in File S1). Single-nucleotide variants (SNVs) were identified that distinguished the three isolates using stringent criteria (see Methods section). This found a total of 54 SNVs, clearly demonstrating the very close relationship between the strains (the average pair of *H. influenzae* isolates differ by ∼40,000 SNVs). 45 of the variants were in coding sequences, and 39 of these caused nonsynonymous changes in 19 different genes (Table 7). This high proportion of nonsynonymous changes strongly suggests that these changes are selectively beneficial. These genes encode proteins belonging to the following functional categories: permease involved in resistance to AMPs and in heme uptake (1), heme/haemoglobin/haptoglobin acquisition (5), LOS biosynthesis (1), outer membrane (1), metabolism (7), transporters (4) (Table 7). Except for three SNVs in a single gene, every SNV was either present in both later isolates or only in isolate NTHi 1c, supporting the hypothesis that NTHi1a is an ancestor of NTHi1b, and NTHi1b is an ancestor of NTHi1c. At *ompP2* (ORF1641), 3 of the 13 SNVs were inconsistent with stepwise mutation accumulation. The most likely explanation is that one of the *ompP2* alleles in NTHi1b and NTHi1c was independently derived by recombination with another *H. influenzae* strain in the patient’s overall population, as was previously reported for NTHi *ompP2* alleles in a COPD patient [8].

**Table 7:** List of open reading frames (ORFs) with single nucleotide variants (SNVs) among isolates NTHi1a, 1b and 1c.

ORF	Alt	NTHi1a	NTHi1b	NTHi1c	aaref	aaalt	aapos	Proteinlength	Genename	HiRdKW20(%aa id)	86-028NP(%aa id)	Function
64	A	0	0	1	E	K	269	297	*ppnK*	HI0072 (99)	NTHI0084 (99)	Inorganic polyphosphate/ATP-NAD kinase
140	G	0	0	1	T	A	162	705	*spoT*	HI1741 (99)	NTHI2052 (99)	Guanosine-3′,5′-bis(di-P) 3′-pyrophosphohydrolase
156	G	0	0	1	D	G	398	996	*hgpC*	Hi0712 (86)	NTHI0840,out of frame	Haemoglobin-haptoglobin binding protein
	A	0	1	1	E	K	433					
	T	0	1	1	T	I	895					
345	T	0	1	1	G	V	96	455	*ompP1*	Hi0401 (90)	NTHI0522 (88)	Long-chain fatty acid ABC transporter
423	A	0	0	1	G	D	400	401		Hi0477 (99)	NTHI0607 (99)	Tyrosine-specific transport protein 1
583	T	0	0	1	A	S	139	658	*cpdB*	Hi0583 (98)	NTHI0741 (98)	2′,3′-cyclic nucleotide 2′-phosphodiesterase/3′-nucleotidase
587	A	0	0	1	T	K	525	756	*hgpB*	Hi0661 (81)	NTHI0782 (86)	Hemoglobin-haptoglobin binding protein B
1001	G	0	1	1	T	A	79	296	*sapC*	Hi1640 (99)	NTHI1399 (99)	ABC transporter permease
1009	A	0	1	1	P	T	280	910	*hup*	Hi1217 (92)	NTHI1390 (90)	Heme utilization protein
	T	0	0	1	D	Y	291					
	G	0	0	1	T	A	555					
1057	A	0	0	1	A	D	255	385	*metX*	Hi1172 (98)	NTHI1340 (99)	S-adenosylmethionine synthetase
1127	A	0	0	1	R	S	85	196	*nudH*	Hi0901 (96)	NTHI1068 (99)	Dinucleoside polyphosphate hydrolase
1140	A	0	1	1	P	T	278	430	*purD*	Hi0888 (98)	NTHI1052 (98)	Phosphoribosylamine-glycine ligase
1146	G	0	0	1	T	A	2	457		Hi0883 (99)	NTHI1046 (99)	Na+/alanine symporter
1153	A	0	0	1	E	K	395	404	*obgE*	HI0877 (95)	NTHI1040 (96)	GTPase
1155	G	0	1	1	G	G	223	435	*pepB*		NTHI1038 (98)	Aminopeptidase
1301	G	0	1	1	P	P	34	516	*hgpB*	Hi0661 (85)	NTHI0782 (82)	Hemoglobin-haptoglobin binding protein B
	C	0	1	1	S	L	40					
	T	0	1	1	S	L	40					
	A	0	1	1	S	L	40					
	C	0	1	1	R	R	44					
	C	0	1	1	Y	H	116					
	A	0	1	1	D	N	320					
1389	T	0	1	1	G	G	164	449	*accC*	Hi0972 (99)	NTHI1145 (99)	Acetyl-CoA carboxylase biotin carboxylase subunit
1448	G	0	0	1	G	G	54	615		Hi1051 (99)	NTHI1212 (99)	Multidrug ABC transporter
1499	T	0	0	1	A	V	346	408		Hi1104 (99)		Transporter protein
1594	C	0	1	1	Q	H	226	242	*kdkA*	Hi0260.1 (97)	NTHI0369 (88)	3-deoxy-D-manno-octulosonic-acid kinase
1596	A	0	1	1	W	*	133	734	*hxuC*	Hi0262 (94)	NTHI0369 (88)	heme-hemopexin utilization protein C
1638	G	0	1	1	R	R	234	489	*guaB*	Hi0221 (99)	NTHI0324 (99)	Inosine 5′-monophosphate dehydrogenase
1641	G	0	0	1	V	G	23	376	*ompP2*	Hi0139 (78)	NTHI0225 (79)	Outer membrane protein
	C	0	1	0	I	T	91					
	C	0	1	1	I	P	91					
	G	0	1	1	E	G	181					
	T	0	0	1	R	I	225					
	A	0	1	1	V	I	228					
	A	0	1	0	S	*	229					
	T	0	0	1	A	S	230					
	T	0	0	1	A	S	233					
	C	0	1	1	T	P	271					
	G	0	1	1	Y	D	273					
	C	0	1	0	T	P	275					
	G	0	1	1	I	R	277					

ORF: open reading frame number in NTHi1a annotation.

Alt: nucleotide showing a SNP.

0: nucleotide identical to the one in strain NTHi1a; 1: SNV, compared to NTHi1a.

aa ref: amino acid in NTHi1a; aa alt: amino acid replacement due to SNV: aa position: replacement location.

ORFs in grey follow the pattern 0-1-1, i.e. ORFs with SNVs in NTHi1b and 1c, when compared to NTHi1a.

To identify candidate variants responsible for AMP resistance, we focused on the nine loci containing coding SNVs present in both NTHi1b and NTHi1c, since these strains have similar AMP resistance (Table 7). A good candidate for the increase in AMP resistance is the T79A change in SapC, a permease translocator of the SapABCDZ system involved in NTHi resistance to AMPs and in heme utilization [62]. Four other genes in this set of nine were iron transporters: *hgpC*, *hup*, *hgpB*, and *hxuC*. We found three amino acid changes in HgpC: D398G, E433K and T895I; three changes in Hup: P280T, D291Y and T555A; and three changes in HgpB: S40L, Y116H and D320N. The change in HxuC is most likely a null mutation due to the early stop codon W133*. Although the consequences of variation in these iron transporters are not known, the multiple changes strongly suggest the action of natural selection. We also found the amino acid change Q226H in the sequence of KdkA, a kinase encharged of 3-deoxy-D-manno-octulosonic acid (Kdo) phosphorylation; the amino acid change G96V in the long-chain fatty acid transporter OmpP1; and the amino acid change P278T in the phosphoribosylamine-glycine ligase PurD.

We pursued the variation observed in the *sapC* and *kdkA* genes, by sequencing these two genes in the isolates recovered from patient 2 and 3. We observed sequence variation among patients (amino acids shown in bold, Figure S4 and Figure S5 in File S1). Clonal isolates within each patient had identical *sapC* and *kdkA* sequences, excluding a possible involvement of SNVs in these two genes in the increasing resistance to AMPs observed for isolates recovered from patients 2 and 3.

In sum, whole genome sequence of the three clonal isolates from patient 1 revealed amino acid changes that could account for the observed increasing resistance to AMPs in isolates NTHi1b and 1c, compared to NTHi1a.

## Discussion

Understanding chronic infection by NTHi associated with obstructive lung disease in adults is relevant due to its implications for the progression of the disease. In this work, we examined a range of phenotypic traits associated with pathogenesis in three series of NTHi clonal isolates recovered from three obstructive lung disease patients. Each series contained two or three isolates with identical PFGE profile, recovered from sputum samples from patients in acute condition during different medical visits. This is, to our knowledge, the first phenotypic study on series of clonal NTHi isolates recovered from adult respiratory patients which is not restricted to COPD. Phenotypes were compared to those shown by a number of newly acquired strains.

Persistent isolates were distinguished by epithelial infection and biofilm growth: they adhered poorly to epithelial cells and did not form biofilms. Adhesion data agree with the observation that *H. influenzae* isolates recovered from COPD exacerbation adhere to airway epithelial cells more strongly than colonisers [12]. *P. aeruginosa* isolates obtained from chronically infected patients also showed a reduced ability to associate with A549 cells [63]. Low adhesion may dampen pathogen-induced inflammatory responses of respiratory epithelial cells, favouring host immunity evasion. This observation may be relevant, given that host inflammation is crucial for the effective clearance of infections. Our data show a limited stimulation of IL-8 secretion by epithelial cells upon infection with NTHi strains. These results agree with a previous report showing that newly acquired NTHi strains infecting the airway during COPD exacerbation induce more IL-8 secretion by epithelial cells than colonizers [12]. NTHi biofilm has also been shown to be a variable feature [64], associated with pediatric nasopharyngeal carriage [65] and CF persistence [66], but not with COPD colonization [12]. Here, we observed that the three series of persistent isolates did not form a detectable biofilm on microfermenters glass slides, unlike the newly acquired isolates. Of note, *P. aeruginosa* biofilm formation by non-mucoid CF isolates follows a trend of decreased adherence with progression of the chronic lung infection [67], and non-attached aggregates have been related to *P. aeruginosa* ability to cause chronic infection [68]. Like epithelial adhesion, biofilm growth is considered to be an important event in early stages of NTHi infection. The relationship between the phenotypic trends observed and persistent infection by NTHi is currently unknown. Self-aggregation clustered isolates by patient. Given that NTHi is well adapted to its human host, self-aggregation could be a host-dependent adaptive phenotype, with unknown implications in pathogenesis.

We observed an increasing resistance to AMPs in the three series of persistent isolates tested. LOS decoration with PCho and lipid A acylation did not account for this observation. All strains rendered a PCho signal, despite the fact that some of them contained a phase OFF *lic1A* gene, according to the number of repeats. Evidence points out that PCho expression may also be influenced by a second *lic1* locus that allows for dual PCho substitutions in the same strain [69], which could also contribute to explain the observations shown in this study. LOS decoration with PCho and lipid A acylation prompted us to further analyse the series of three strains recovered from patient 1. Genome sequence revealed that these three isolates are not identical, despite displaying the same PFGE profile. Given that increasing resistance to AMPs was observed for NTHi1b and 1c, compared to NTHi1a, we considered NTHi1a as a reference and looked for SNVs present in both NTHi1b and 1c. We found one SNV in the *sapC* gene, which belongs to the *sapABCDZ* operon, encoding a system involved in NTHi resistance to AMPs. SapA, located at the periplasm, binds AMP molecules that are translocated to the bacterial cytoplasm through the SapBC translocon. Once in the cytosol, AMP molecules are degraded. Whether the replacement SapCT79A accounts for the observed increasing resistance to AMPs in this series of isolates is currently unknown. SapABCDZ is also involved in heme uptake [62]. *H. influenzae* does not synthesise heme. To overcome this disability, it is endowed with several heme uptake systems [70]. Together with *sapC*, we found polymorphisms in the heme acquisition systems *hgpB*, *hgpC*, *hup* and *hxuC*. Given that SapABCDZ conjugates AMPs resistance and heme uptake, and based on the sequence data presented here, we speculate about a potential crosstalk between bacterial systems responsible for AMPs resistance and for heme acquisition. Although formally different, *Staphylococcus aureus* small colony variants (SCVs), which are impaired in heme biosynthesis, show reduced susceptibility to the AMP lactoferricin B [71]. We should also consider the observed SNVs in genes encoding heme uptake systems as possible adaptations to overcome host nutritional immunity. In fact, restriction of heme-iron in the host may foster long-term phenotypic changes that better equip NTHi for survival at infection sites [72]. Whether there is a relationship between phenotypic changes linked to nutritional immunity and genome changes is unknown. Clonal adaptation to iron limitation has been reported for *Burkholderia cenocepacia* [73], which, in that case, may be related to up-regulation of genes involved in iron uptake [74].

We also found a SNV in the Kdo kinase gene *kdkA*. The amino acid sequence motif of the active site of KdkA has been elucidated previously [75], and does not include the amino acid position 226. We lack evidence for KdkA involvement in NTHi resistance to AMPs. However, disruption of *kdkA* in *Pasteurella multocida* increases bacterial sensitivity to the bactericidal peptide fowlicidin [76]. In addition, we found a SNV in the long chain fatty acid transporter encoding *ompP1*/*fadL* gene. Interestingly, premature termination of *H. influenzae fadL* has been previously found to confer resistance to the antibacterial compound A-344583 [77]. Finally, we found a SNV in the phosphoribosylamine glycine ligase encoding gene *purD*. We lack evidence for NTHi purine biosynthesis relationship with AMP resistance. However, *S. aureus* strains resistant to vancomycin revealed increased transcription of up to 15 genes involved in purine biosynthesis or transport [78].

Several reports have linked variation in phase variable genes to NTHi adaptation or persistence: increasing number of single sequence repeats in *hmw1A* and *hmw2A* promoter regions linked to diminishing expression of HMW1A and HMW2A [9]; *lic2A*, *lgtC* and *lex2A* switch from phase-off to phase-on following serial passage in human serum, reducing binding of bactericidal antibody to LOS [79]; *licA* and *igaB* switch from phase-off to phase-on during experimental human nasopharynx colonization by NTHi [80]. Differently, this is, to our knowledge, the first study on clonal NTHi isolates where diversity has been assessed at the genomic level, revealing a repertoire of newly found SNVs that could be relevant for NTHi infection and persistence.

This investigation was performed on stored material, using only a single colony from each original growth culture, so we cannot exclude the presence of multiple *H. influenzae* strains in these cultures. In COPD, both simultaneous presence of multiple *H. influenzae* and the presence of one strain of *H. influenzae* in sputum samples have been reported [7], [81]. We acknowledge this limitation in the present study.

In summary, we provide clues to nontypable *H. influenzae* adaptation during infection. Due to its clinical relevance, a comprehensive understanding of microbial traits associated with NTHi persistence may help to establish guidelines applicable in the prevention or treatment of chronic lung disease exacerbations. Also, we found changes at the genome level that could account for NTHi adaptation. Genome sequencing of sets of isolates sequentially cultured from chronically infected patients has revealed genome evolution and genetic adaptation for several bacterial pathogens, including *P. aeruginosa*, *Burkholderia dolosa* or *Helicobacter pylori* [82]–[85]. Further application of genome sequence-based systematic approaches to long series of clonal variants will deliver valuable information on NTHi adaptive strategies that may offer new opportunities for treatment of NTHi infection in chronic respiratory patients.

## Supporting Information

File S1 **This file contains Figures S1 to S5.** Figure S1. Characterization of serial clonal isolates recovered from patient 1, NTHi1a, 1b and 1c. (A) Analysis of *Haemophilus influenzae* lipid A. Representative negative ion MALDI-TOF lipid A mass spectra of the different samples analyzed. Right panel: proposed structures of the main molecular species present obtained. All strains tested, both in the absence or in the presence of sub-lethal [PxB], displayed identical lipid A structure. (B) Growth of NTHi1a, 1b, 1c and Rd KW20 in rich medium. Cells were grown in sBHI broth in a BioScreen incubator with the OD_600 nm_ recorded every 10 min. Each line shows the mean of 8 to 12 replicates for one strain. Optical densities lower than 0.01 are not shown. To account for differences in inoculum sizes, the lines have been shifted along the *x* axis to superimpose the growth curves of all strains. Color code: NTHi1a, red; 1b, yellow; 1c, green; Rd KW20, blue. (C) Transformation frequencies of *H. influenzae* strains NTHi1a, 1b and 1c. Vertical bars represent the means of at least three biological replicates ± standard deviations. We did not observe significant differences. (D) Bacterial loads in the lungs of CD1 mice infected by NTHi1a, 1b and 1c. Mice were infected intranasally with 10^8^ bacteria. Bacterial counts in lungs at 24 or 48 h PI were determined. Results are reported as log_10_ CFU/lung. Lines inside boxes represent median values. Statistical differences could not be observed among isolates NTHi1a, 1b and 1c. In the three cases, bacterial recovery at 48 (white box) was significantly lower than at 24 (grey box) h PI (p<0.0001). Figure S2. Control assembly. Mauve alignment visualized after contig reordering between the reference genome 86-028NP (top row) and the RR3131 draft assembly (bottom row). The x-axis indicates genome coordinate; the y-axis shows % nucleotide identity. The vertical red lines indicate contig boundaries. Because the genomes are circular, a single “inversion” is detected at the chromosome “ends” (green line). The six white gaps in the top row indicate the six rDNA repeats in 86-028NP. Figure S3. Synteny between the clinical isolates NTHi1a, 1b and 1c. Mauve alignment visualized after iterative contig reordering between the three clinical isolates. Rows from top to bottom are NTHi1a, NTHi1b, and NTHi1c. The x-axis indicates genome coordinate; the y-axis indicates % nucleotide identity. The vertical red lines indicate contig boundaries. The white gap in NTHi1c represents 28 kb missing from the assemblies of the others and likely indicates a novel phage insertion. Figure S4. CLUSTAL 2.1 multiple sequence alignment of SapC protein variants from patients 1, 2 and 3. Color code: patient 1, red (T79A replacement in NTHi1b and 1c is highlighted in turquoise); patient 2, blue; patient 3, black. SNV are indicated in bold colored. Figure S5. CLUSTAL 2.1 multiple sequence alignment of KdkA protein variants from patients 1, 2 and 3. Color code: patient 1, red (Q226H replacement in NTHi1b and 1c is highlighted in turquoise); patient 2, blue; patient 3, black. SNV are indicated in bold colored.
(PDF)
