# Correction: Characterization of Nontypable *Haemophilus influenzae* Isolates Recovered from Adult Patients with Underlying Chronic Lung Disease Reveals Genotypic and Phenotypic Traits Associated with Persistent Infection

**DOI:** 10.1371/journal.pone.0107686

**Published:** 2014-09-03

**Authors:** The PLOS ONE Staff

Canada Foundation for Innovation is also a funder for this study. Please refer to the correct funding and acknowledgement statements below:

Funding:

JCM was supported by a postdoctoral fellowship from the U.S. National Institutes of Health (5F32AI084427). This work has been funded by grants from ISCIII PS09/00130, Ministerio Economía y Competitividad (MINECO) SAF2012-31166, Dpto. Salud Gobierno Navarra 359/2012, and Ministerio

Educación PRX 12/00191 to JG, PS09/01904 to JL, and Canadian Institute of Health Research to RJR. CIBERES is an initiative from ISCIII, Spain. Sequencing was performed at the Pharmaceutical Sciences Sequencing Centre at the University of British Columbia, supported by grants from the CFI (Canada Foundation for Innovation) and the faculty of Pharmaceutical Sciences. The funders had no role in study design, data collection and analysis, decision to publish, or preparation of the manuscript.

Acknowledgements:

We thank Javier Moleres and Antonio López-Gómez for their technical help. We thank Sunita Sinha and Corey Nislow for library preparation and next-generation sequencing, performed at the Pharmaceutical Sciences Sequencing Centre at the University of British Columbia, and supported by grants from the Canada Foundation for Innovation and the faculty of Pharmaceutical Sciences to C. Nislow. We thank Christopher J. Grassa for critical help with the genome assembly pipeline.

## References

[1] Garmendia J, Viadas C, Calatayud L, Mell JC, Martí-Lliteras P, et al (2014) Characterization of Nontypable Haemophilus influenzae Isolates Recovered from Adult Patients with Underlying Chronic Lung Disease Reveals Genotypic and Phenotypic Traits Associated with Persistent Infection. PLoS ONE 9(5): e97020 doi:10.1371/journal.pone.0097020 24824990 10.1371/journal.pone.0097020 PMC4019658

