# Supplementary material for: Characterization of Nontypable Haemophilus influenzae Isolates Recovered from Adult Patients with Underlying Chronic Lung Disease Reveals Genotypic and Phenotypic Traits Associated with Persistent Infection
Source: PLoS One. 2014 May 13;9(5):e97020. doi: 10.1371/journal.pone.0097020 (PMC4019658; doi:10.1371/journal.pone.0097020)
Supplement: File S1 — This file contains Figures S1 to S5. Figure S1. Characterization of serial clonal isolates recovered from patient 1, NTHi1a, 1b and 1c. (A) Analysis of Haemophilus influenzae lipid A. Representative negative ion MALDI-TOF lipid A mass spectra of the different samples analyzed. Right panel: proposed structures of the main molecular species present obtained. All strains tested, both in the absence or in the presence of sub-lethal [PxB], displayed identical lipid A structure. (B) Growth of NTHi1a, 1b, 1c and Rd KW20 in rich medium. Cells were grown in sBHI broth in a BioScreen incubator with the OD600 nm recorded every 10 min. Each line shows the mean of 8 to 12 replicates for one strain. Optical densities lower than 0.01 are not shown. To account for differences in inoculum sizes, the lines have been shifted along the x axis to superimpose the growth curves of all strains. Color code: NTHi1a, red; 1b, yellow; 1c, green; Rd KW20, blue. (C) Transformation frequencies of H. influenzae strains NTHi1a, 1b and 1c. Vertical bars represent the means of at least three biological replicates ± standard deviations. We did not observe significant differences. (D) Bacterial loads in the lungs of CD1 mice infected by NTHi1a, 1b and 1c. Mice were infected intranasally with 108 bacteria. Bacterial counts in lungs at 24 or 48 h PI were determined. Results are reported as log10 CFU/lung. Lines inside boxes represent median values. Statistical differences could not be observed among isolates NTHi1a, 1b and 1c. In the three cases, bacterial recovery at 48 (white box) was significantly lower than at 24 (grey box) h PI (p<0.0001). Figure S2. Control assembly. Mauve alignment visualized after contig reordering between the reference genome 86-028NP (top row) and the RR3131 draft assembly (bottom row). The x-axis indicates genome coordinate; the y-axis shows % nucleotide identity. The vertical red lines indicate contig boundaries. Because the genomes are circular, a single “inversion” is detec [file pone.0097020.s001.pdf]

Supporting Information

Table S1. P5 sequence in predicted extracellular loops 1 to 5 for representative NTHi isolates.

		NTHi isolate														
		Patient 1			Patient 2		Patient 3			Patient 4		Patient 5				Patient 6
**P5**	**Loop sequence**	**1a**	**1b**	**1c**	**2a**	**2b**	**3a**	**3b**	**3c**	**4a**	**4b**	**5a**	**5b**	**5c**	**5d**	**6a**
**Loop1**	GQASFHDGVHALHDGVHALARQNKAGYHRNS							x	x							
	GQGSFHDGINNNGAIKQDLEL--GYGHRRNT	x	x	x	x	x										
	GQGSFHDGINNNGAIKENLTS--GYGYRRNT						x									
	GQGSFHDGINNNGAIQKDLEL--GYGYRRNT												x	x		
	GQGSFHDGINRNRVIIDGLPGDYG---RRNT											x				
	GQGSFHDGINNNGAIKNNLPGY--YGYRRNT									x						
	GQASFHDGINNNGAIKKDLLGLASYGYRRNT										x					
	GQASFHDGLRALARENKV-------GYHRNS														x	
	GQGSFHDGINNNGAIKQNLSSAN-YGYRRNT															x
**Loop2**	DNFGRAKLRSNGQALAKHTN						x									
	DDFGRAKFRVAGKPKVKHTN							x	x							
	DNFGRAKLREAGKPRAKHTN	x	x	x	x	x										
	DDFGRAKGREKGRTVAKHTN												x	x		
	DNFGRVKARQERKTLAKHTN									x		x				
	DDFGRVKLRLAGKPKVKHTN										x					
	DDFGRAKGREKGKTVAKHTN														x	
	DNFGRVKLREAGKPKAKHTN															x
**Loop3**	VRSDYKFY----GTRDHKKGR							x	x							
	VRSDYKFYEVANGTRERKKGR									x		x				
	VRSDYKVYEVADGTRDHKKGR						x									
	VRSDYKFYADANGTRDHKKGR	x	x	x	x	x										
	VRSDYKLYNKNSSTLKDLGEH													x		
	VRSDYKFYDKNGSTFKNPGEH												x			
	VRSDYKFYEDANGTRDHKKGR										x					
	VRSDYKLYNKNSSSNSTLKNL														x	
	VRSDYK-YEVANGTRDHKKGR															x
**Loop4**	LTRVGKYRAQDK						x									
	LTRVGKYRPQDK	x	x	x	x	x		x	x		x		x	x		x
	LTRVGKYRTQKT														x	
	LTRVGKFRTQDK									x		x				
**Loop5**	RFGQGAAP-VVAAPEVVSKT	x	x	x	x	x	x	x	x	x	x	x	x			
	RFGQGEAP-VVAAPEMVSKT															x
	RFGQ-AAP-VVAAPEMVSKT													x		
	RFGQGAAP-VVAAPEMVSKT														x	

Table S2. Self-aggregation of NTHi strains (t=h).

Patient	Strain	0	1	1.5	2	3	4	5	6	7
	E. coli C600	1,03±0,04	0,99±0,05	0,97±0,04	0,97±0,03	0,99±0,03	0,98±0,02	0,98±0,03	1±0,04	0,990±05
1	NTHi1a	1,02±0,05	0,50±0,06[b]	0,25±0,06[b]	0,14±0,06	0,07±0,03	0,06±0,01	0,04±0,01	0,03	0,03
	NTHi1b	1,06±0,03	0,54±0,10[b]	0,31±0,12[b]	0,17±0,09	0,08±0,02	0,06±0,01	0,04	0,04	0,04
	NTHi1c	1,01± 0,08	0,45±0,09[b]	0,21±0,02[b]	0,1±0,04	0,05± 0,02	0,04	0,04	0,04±0,01	0,02
2	NTHi2a	1,02±0,03	0,30±0,07	0,14±0,04	0,06±0,03	0,04±0,01	0,03±0,01	0,03±0,02	0,03±0,02	0,02±0,01
	NTHi2b	1,04±0,02	0,27±0,15	0,1±0,05	0,06±0,01	0,04±0,02	0,03±0,02	0,02±0,02	0,03±0,02	0,02±0,01
3	NTHi3a	1,03±0,04	1,01±0,05[a]	1,04±0,02[a]	0,98±0,05[a]	0,94±0,07[a]	0,98±0,06[a]	0,92±0,1[a]	0,88±0,08[a]	0,89±0,07[a]
	NTHi3b	1,04±0,04	1,02±0,05[a]	1±0,06[a]	1,02±0,07[a]	0,92±0,08[a]	0,89±0,18[a]	0,86±0,23[a]	0,84±0,18[a]	0,85±0,22[a]
	NTHi3c	1,02±0,06	1,03±0,03[a]	1,04±0,06[a]	1,02±0,04[a]	0,96±0,02[a]	0,96±0,04[a]	0,94±0,03[a]	0,92±0,03[a]	0,93±0,03[a]
5	NTHi5b	1,05±0,01	0,41±0,08	0,20±0,02	0,11±0,02	0,07±0,01	0,05±0,02	0,03±0,02	0,02±0,02	0,02±0,02
6	NTHi6a	1,03±0,04	0,83±0,11[c]	0,58±0,17[c]	0,33±0,14	0,13±0,04	0,08±0,02	0,05±0,01	0,04±0,02	0,03±0,02

[a]OD_{600nm} values for series isolated from patient 3 were significantly higher ($p<0.0001$ from 1 h to the end of the assay) than those obtained for the other NTHi strains tested.
[b]OD_{600nm} values for NTHi1a, 1b and 1c were significantly higher than those obtained for NTHi2a and 2b at 1 ($p<0.0001$) and 1,5 h ($p<0.001$).
[c]NTHi6a presented OD_{600nm} values higher than those obtained for strains from patients 1 and 2 at 1-1.5 h ($p<0.0001$).

Table S3. Lipid A structures identified in the NTHi isolates analyzed.

$[M-H]^-$	Fatty acid substitutions			
	C3’	C2’	C3	C2
1824	14:0[3-O(14:0)]	14:0[3-O(14:0)]	14:0(3-OH)	14:0(3-OH)
1744	14:0[3-O(14:0)]	14:0[3-O(14:0)]	14:0(3-OH)	14:0(3-OH)
1388	OH	14:0[3-O(14:0)]	14:0(3-OH)	14:0(3-OH)

Table S4. Summary statistics for draft genome assemblies.

**Features**	**RR3131**	**NTHi1a**	**NTHi1b**	**NTHi1c**
# of reads	2,349,021	13,668,344	3,420,997	6,535,653
% overlapped	19.7%	40.1%	39.9%	29.4%
# contigs	21	23	17	16
total length (bp)	1,891,683	1,787,944	1,794,570	1,803,206
N50 (bp)	198,461	224,231	246,701	247,076
median (bp)	36,044	24,844	38,138	51,313
largest contig (bp)	414,414	507,594	426,945	508,640

Figure S1

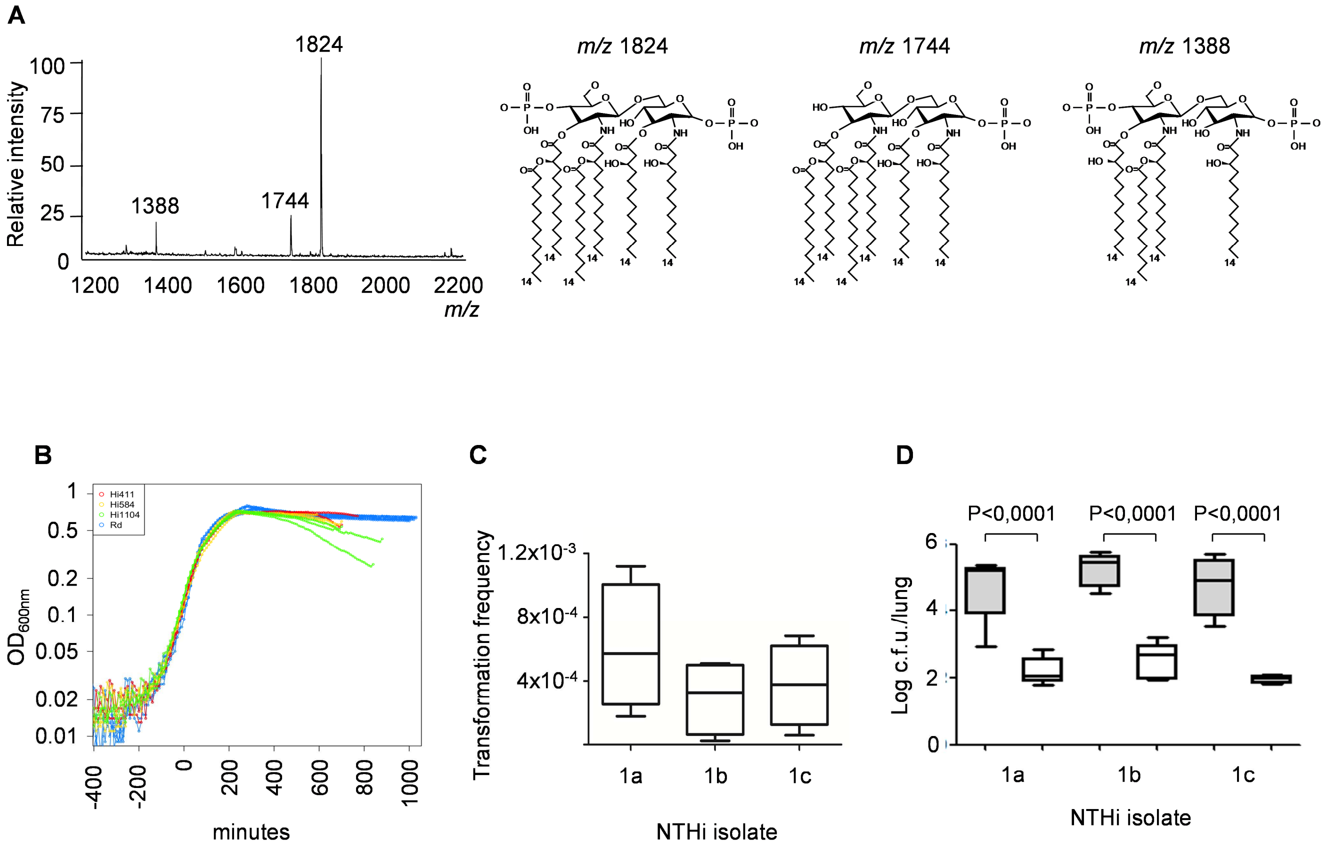
A
1824
1388
1744
Relative intensity
m/z
m/z 1824
m/z 1744
m/z 1388
B
OD600nm
minutes
Hi411
Hi584
Hi1104
Rd
C
Transformation frequency
NTHi isolate
D
P<0,0001
Log c.f.u./lung
NTHi isolate
1a
1b
1c

Figure S2

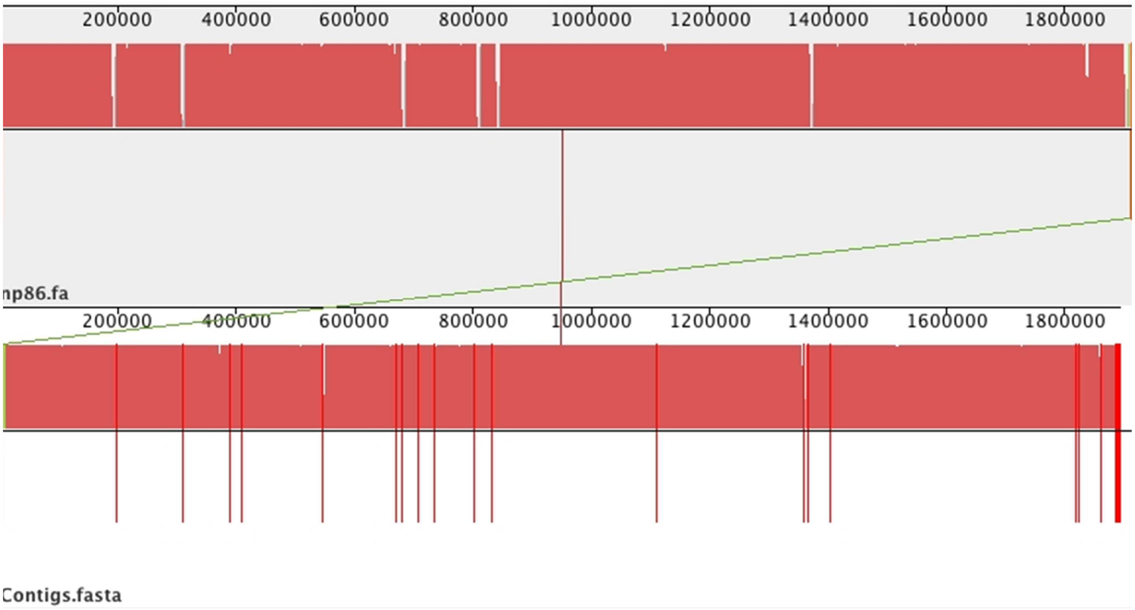

200000
400000
600000
800000
1000000
1200000
1400000
1600000
1800000
np86.fa
200000
400000
600000
800000
1000000
1200000
1400000
1600000
1800000
Contigs.fasta

Figure S3

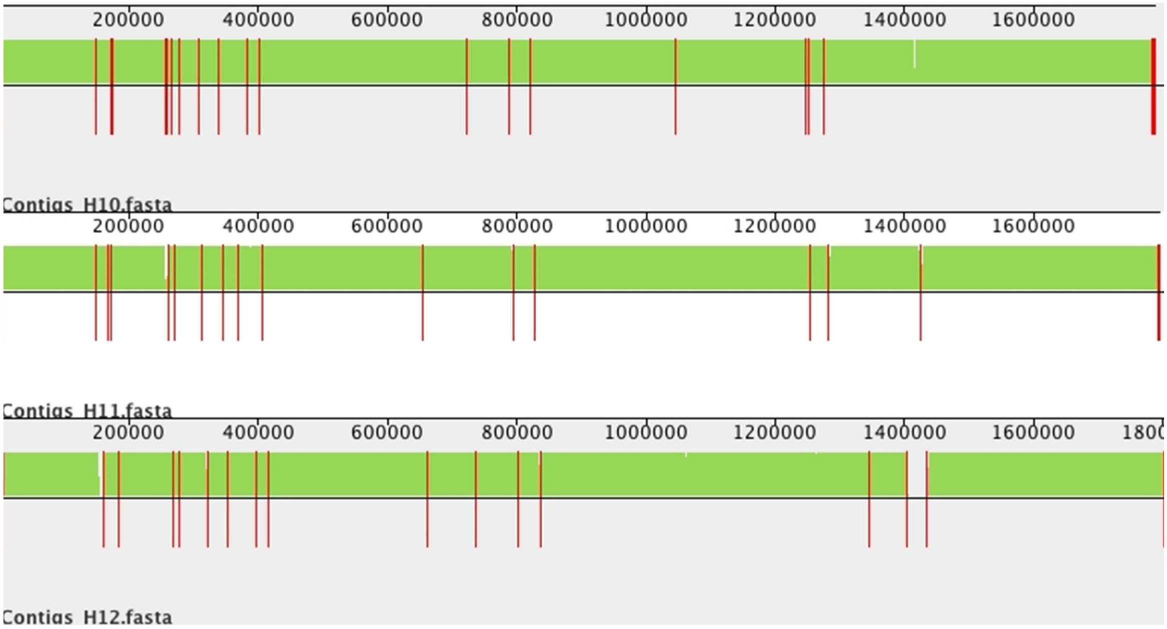
200000
400000
600000
800000
1000000
1200000
1400000
1600000
Contigs_H10.fasta
Contigs_H11.fasta
Contigs_H12.fasta

Figure S4

```
NTHi2a SapC     MQNKEPDEFRESTSIFQIWLRFRQNTIALFSFYLLIALIFTALFASYLAPYADNRQFIGQ 60
NTHi2b          MQNKEPDEFRESTSIFQIWLRFRQNTIALFSFYLLIALIFTALFASYLAPYADNRQFIGQ 60
NTHi1c          MQNKEPDEFRESTSIFQIWLRFRQNTIALFSFYLLIALIFTALFASYLAPYADNRQFIGQ 60
NTHi1b          MQNKEPDEFRESTSIFQIWLRFRQNTIALFSFYLLIALIFTALFASYLAPYADNRQFIGQ 60
NTHi1a          MQNKEPDEFRESTSIFQIWLRFRQNTIALFSFYLLIALIFTALFASYLAPYADNRQFIGQ 60
NTHi3a          MQNKEPDEFRESTSIFQIWLRFRQNTIALFSFYLLIALIFTALFASYLAPYADNRQFIGQ 60
NTHi3b          MQNKEPDEFRESTSIFQIWLRFRQNTIALFSFYLLIALIFTALFASYLAPYADNRQFIGQ 60
NTHi3c          MQNKEPDEFRESTSIFQIWLRFRQNTIALFSFYLLIALIFTALFASYLAPYADNRQFIGQ 60
                ************************************************************

NTHi2a          ELMPPSWVDRGKIAFFFGADDLGRDILSRLIMGTRYTLGSALLVVFSVAIIGGALGIIAG 120
NTHi2b          ELMPPSWVDRGKIAFFFGADDLGRDILSRLIMGTRYTLGSALLVVFSVAIIGGALGIIAG 120
NTHi1c          ELMPPSWVDRGKIAFFFGADDLGRDILSRLIMGTRYTLGSALLVVFSVAIIGGALGIIAG 120
NTHi1b          ELMPPSWVDRGKIAFFFGADDLGRDILSRLIMGTRYTLGSALLVVFSVAIIGGALGIIAG 120
NTHi1a          ELMPPSWVDRGKIAFFFGTDDLGRDILSRLIMGTRYTLGSALLVVFSVAIIGGALGIIAG 120
NTHi3a          ELMPPSWVDRGKIAFFFGTDDLGRDILSRLIMGTRYTLGSALLVVFSVAIIGGALGIIAG 120
NTHi3b          ELMPPSWVDRGKIAFFFGTDDLGRDILSRLIMGTRYTLGSALLVVFSVAIIGGALGIIAG 120
NTHi3c          ELMPPSWVDRGKIAFFFGTDDLGRDILSRLIMGTRYTLGSALLVVFSVAIIGGALGIIAG 120
                ******************:*****************************************

NTHi2a          LLKGIKARFVGHIFDAFLSLPILLIAVVISTLMEPSLWNAMFATLLAILPYFIHTIYRAI 180
NTHi2b          LLKGIKARFVGHIFDAFLSLPILLIAVVISTLMEPSLWNAMFATLLAILPYFIHTIYRAI 180
NTHi1c          LLKGIKARFVGHIFDAFLSLPILLIAVVISTLMEPSLWNAMFATLLAILPYFIHTIYRAI 180
NTHi1b          LLKGIKARFVGHIFDAFLSLPILLIAVVISTLMEPSLWNAMFATLLAILPYFIHTIYRAI 180
NTHi1a          LLKGIKARFVGHIFDAFLSLPILLIAVVISTLMEPSLWNAMFATLLAILPYFIHTIYRAI 180
NTHi3a          LLKGIKARFVGHIFDAFLSLPILLIAIVISTLMEPSLWNAMFATLLAILPYFIHAIYQAI 180
NTHi3b          LLKGIKARFVGHIFDAFLSLPILLIAIVISTLMEPSLWNAMFATLLAILPYFIHAIYQAI 180
NTHi3c          LLKGIKARFVGHIFDAFLSLPILLIAIVISTLMEPSLWNAMFATLLAILPYFIHAIYQAI 180
                **************************:***************************:**:**

NTHi2a          QKELEKDYVVMLKLEGISNQALLKSTILPNITVIYIQEVAHAFVIAVLDISALSFISLGA 240
NTHi2b          QKELEKDYVVMLKLEGISNQALLKSTILPNITVIYIQEVAHAFVIAVLDISALSFISLGA 240
NTHi1c          QKELEKDYVVMLKLEGISNQALLKSTILPNITVIYIQEVAHAFVIAVLDISALSFISLGA 240
NTHi1b          QKELEKDYVVMLKLEGISNQALLKSTILPNITVIYIQEVAHAFVIAVLDISALSFISLGA 240
NTHi1a          QKELEKDYVVMLKLEGISNQALLKSTILPNITVIYIQEVAHAFVIAVLDISALSFISLGA 240
NTHi3a          QQELKKDYVLMLKLEGISNWELLKSTILPNITVIYIQEVARAFVIAVLDISALSFISLGA 240
NTHi3b          QQELKKDYVLMLKLEGISNWELLKSTILPNITVIYIQEVARAFVIAVLDISALSFISLGA 240
NTHi3c          QQELKKDYVLMLKLEGISNWELLKSTILPNITVIYIQEVARAFVIAVLDISALSFISLGA 240
                *:**:****:*********  *******************:*******************

NTHi2a          QRPTPEWGAMIKDSLELLYLAPWTVLLPGFAIIFTILLSIIFSNGLTKAINQHQE 295
NTHi2b          QRPTPEWGAMIKDSLELLYLAPWTVLLPGFAIIFTILLSIIFSNGLTKAINQHQE 295
NTHi1c          QRPTPEWGAMIKDSLELLYLAPWTVLLPGFAIIFTILLSIIFSNGLTKAINQHQE 295
NTHi1b          QRPTPEWGAMIKDSLELLYLAPWTVLLPGFAIIFTILLSIIFSNGLTKAINQHQE 295
NTHi1a          QRPTPEWGAMIKDSLELLYLAPWTVLLPGFAIIFTILLSIIFSNGLTKAINQHQE 295
NTHi3a          QRPTPEWGAMIRDSLELLYLAPWTVLLPGFAIIFTILLSIIFSNGLTKTINQHQE 295
NTHi3b          QRPTPEWGAMIRDSLELLYLAPWTVLLPGFAIIFTILLSIIFSNGLTKTINQHQE 295
NTHi3c          QRPTPEWGAMIRDSLELLYLAPWTVLLPGFAIIFTILLSIIFSNGLTKTINQHQE 295
                ***********:************************************:******
```

Figure S5

```
NTHi1b KdkA MHQFQQDNQYFIFNFDRTFEQATEFFQAEFWQKQERVIGSAKGRGTTYFLQTEDWFGVNC 60
NTHi1c      MHQFQQDNQYFIFNFDRTFEQATEFFQAEFWQKQERVIGSAKGRGTTYFLQTEDWFGVNC 60
NTHi1a      MHQFQQDNQYFIFNFDRTFEQATEFFQAEFWQKQERVIGSAKGRGTTYFLQTEDWFGVNC 60
NTHi2a      MHQFQQDNQYFIFNFDRTFEQATEFFQAEFWQKQERVIGSAKGRGTTYFLQTEDWFGVNC 60
NTHi2b      MHQFQQDNQYFIFNFDRTFEQATEFFQAEFWQKQERVIGSAKGRGTTYFLQTEDWFGVNC 60
NTHi3b      MHQFQQDNQYFIFNFDRTFDQATEFFQAEFWQKQERLIGSAKGRGITYFLQTEDWFGVNC 60
NTHi3c      MHQFQQDNQYFIFNFDRTFDQATEFFQAEFWQKQERLIGSAKGRGITYFLQTEDWFGVNC 60
            *******************:****************:******** **************

NTHi1b      ALRHYYRGGLWGKLNKDRYRFSALDTTRSFAEFHLLQRLYEAGLPVPKPIAARIQKGKLG 120
NTHi1c      ALRHYYRGGLWGKLNKDRYRFSALDTTRSFAEFHLLQRLYEAGLPVPKPIAARIQKGKLG 120
NTHi1a      ALRHYYRGGLWGKLNKDRYRFSALDTTRSFAEFHLLQRLYEAGLPVPKPIAARIQKGKLG 120
NTHi2a      ALRHYYRGGLWGKLNKDRYRFSALDTTRSFAEFHLLQRLYEAGLPVPKPIAARIQKGKLG 120
NTHi2b      ALRHYYRGGLWGKLNKDRYRFSALDTTRSFAEFHLLQRLYEAGLPVPKPIAARIQKGKLG 120
NTHi3b      ALRHYYRGGLWGKLNKDRYRFSALETTRSFAEFHLLQRLYEAGLPVPKPIAARIQKGKLG 120
NTHi3c      ALRHYYRGGLWGKLNKDRYRFSALETTRSFAEFHLLQRLYEAGLPVPKPIAARIQKGKLG 120
            ************************:***********************************

NTHi1b      ICYQADILTEKIENAQDLTALLQTQTLPKETWRQIGRLIRKLHDLQICHTDLNAHNILLQ 180
NTHi1c      ICYQADILTEKIENAQDLTALLQTQTLPKETWRQIGRLIRKLHDLQICHTDLNAHNILLQ 180
NTHi1a      ICYQADILTEKIENAQDLTALLQTQTLPKETWRQIGRLIRKLHDLQICHTDLNAHNILLQ 180
NTHi2a      ICYQADILTEKIENAQDLTALLQTQTLPKETWRQIGRLIRKLHDLQICHTDLNAHNILLQ 180
NTHi2b      ICYQADILTEKIENAQDLTALLQTQTLPKETWRQIGRLIRKLHDLQICHTDLNAHNILLQ 180
NTHi3b      ICYQADILTEKIENAQDLTALLQTQTLPKETWTQIGRLIRKLHDLQICHTDLNAHNILLQ 180
NTHi3c      ICYQADILTEKIENAQDLTALLQTQTLPKETWTQIGRLIRKLHDLQICHTDLNAHNILLQ 180
            ******************************** ***************************

NTHi1b      QAEQEQKCWLLDFDKCGEKSGDFWKAQNLNRLKRSFEKEVGRMNIHFTEQNWADLTAAYHQ 241
NTHi1c      QAEQEQKCWLLDFDKCGEKSGDFWKAQNLNRLKRSFEKEVGRMNIHFTEQNWADLTAAYHQ 241
NTHi1a      QAEQEQKCWLLDFDKCGEKSGDFWKAQNLNRLKRSFEKEVGRMNIQFTEQNWADLTAAYHQ 241
NTHi2a      QAEQEQKCWLLDFDKCGEKSGDFWKAQNLNRLKRSFEKEVGRMNIQFTEQNWADLTSAYHQ 241
NTHi2b      QAEQEQKCWLLDFDKCGEKSGDFWKAQNLNRLKRSFEKEVGRMNIQFTEQNWADLTSAYHQ 241
NTHi3b      QAEQEQKCWLIDFDKCGKKSGDFWKVQNLNRLKRSFEKEVGRMNIQFTEQNWADLTSAYHQ 241
NTHi3c      QAEQEQKCWLIDFDKCGKKSGDFWKVQNLNRLKRSFEKEVGRMNIQFTEQNWADLTSAYHQ 241
            **********:******:*******.*******************:**********:****
```
